# De Novo Synthesis in Cancer Immunotherapy: Applications and Prospects

**DOI:** 10.1002/mco2.70847

**Published:** 2026-07-13

**Authors:** Yang Jin, Yanfeng Wu, Jianhua Luo

**Affiliations:** ^1^ Basic Medical Science College Naval Medical University Shanghai PR China; ^2^ National Key Laboratory of Immunity and Inflammation & Institute of Immunology College of Basic Medical Sciences Naval Medical University Shanghai PR China

**Keywords:** adoptive cellular therapy, cancer immunotherapy, cytokine engineering, de novo protein design

## Abstract

De novo protein design enables the creation of proteins with entirely novel structures and biological functions from scratch, offering a promising new paradigm for cancer immunotherapy. Emerging methodologies are integrating principles of rational design, including specificity, modularity, and adaptability, into the development process from the outset. This innovative strategy holds the potential to advance how people understand and intervene against malignancy, through the strategic utilization of immune checkpoint regulation, cytokine engineering, cellular therapy, and signaling pathway remodeling, with each approach demonstrating distinct mechanisms to augment antitumor immune responses. To further optimize these strategies, advanced computational tools and synthetic biology techniques are now being actively deployed to rationally design immune‐modulatory proteins for enhanced cytokine engineering, and to significantly enhance the efficacy of cellular immunotherapies through precision‐engineered chimeric antigen receptor T‐cell immunotherapy constructs and bispecific antibodies, collectively addressing the key limitations of current therapeutic approaches. To this end, this review discusses how the latest advances in these techniques can deepen our understanding of the usage of *de novo*‐designed proteins in cancer immunotherapy, while also highlighting the key limitations that persist in the field.

## Graphical abstract

De novo protein design creates novel structures for cancer immunotherapy, integrating rational design principles like modularity and specificity. Leveraging computational tools, it optimizes cytokine engineering, adoptive cellular therapy, and checkpoint regulation to enhance antitumor immunity. This review discusses how precision‐engineered proteins address current limitations, highlighting recent technical advances, signaling remodeling, and future challenges in clinical translation.

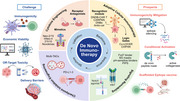

## 1 Introduction

The progress of immunotherapy is primarily attributed to a systemic understanding of tumor‐immune interaction mechanisms and the enhanced capability for druggable modulation, encompassing but not limited to protein signaling pathways. Proteins maintain immune homeostasis via mechanisms including antibodies, cytokines, and receptor–ligand recognition [1, 2]. For instance, protein‐based immune interventions, exemplified by the application of monoclonal antibodies to blockade checkpoint pathways like PD‐1/PD‐L1 or CTLA‐4, have been shown to reverse T‐cell inhibition and restore antitumor activity. These therapeutic approaches have resulted in significantly increased objective response rates (ORR) across various malignancies, including melanoma and non–small cell lung cancer (NSCLC) [1, 3, 4, 5, 6]. Compared with conventional chemotherapy and radiotherapy, this approach demonstrates a distinctive, durable response in a subset of patients, leading to prolonged median overall survival. Furthermore, the exogenous administration of cytokines, such as interleukins (e.g., IL‐2) and interferons (e.g., IFN‐γ), allows for the direct regulation of immune cell differentiation and pro‐inflammatory responses [7, 8]. These clinical advances highlight the importance of protein‐mediated immune regulation and have stimulated extensive efforts to develop next‐generation protein‐based immunotherapeutic strategies.

However, despite these advances, significant challenges remain that limit therapeutic efficacy and patient response rates across broader clinical settings. One major limitation lies in the lack of precise, programmable, and structurally optimized molecular designs capable of effectively addressing the complexity of the tumor microenvironment (TME). Most critically, contemporary interventions often lack the requisite spatiotemporal specificity for localized activation under specific physiological conditions—such as the hypoxia or acidosis characteristic of the TME [9]. This limitation is fundamentally driven by the fact that the TME is not merely a cellular assembly but a sophisticated ecological niche comprising stromal elements and the extracellular matrix (ECM). Within this niche, the tumor orchestrates immune evasion by deploying inhibitory cytokines, such as TGF‐β and IL‐10, to neutralize immune surveillance [10, 11, 12]. Furthermore, the intrinsic heterogeneity of disease models frequently precludes the identification of ideal, universally expressed therapeutic targets, further complicating the development of precision‐guided immunotherapies.

Addressing these multidimensional barriers, therefore, requires a new generation of molecular architectures capable of conditional and environment‐responsive activation. Such molecules should function as environment‐responsive “logic gates” rather than constitutive binders, enabling signaling pathways to be activated only within defined therapeutic windows. Recent advances in synthetic biology, protein engineering, and bioinformatics are now enabling the development of precise, programmable, and tunable protein systems to meet this need [13, 14].

Nevertheless, traditional protein engineering, which largely relies on the modification of existing natural scaffolds (e.g., directed evolution), is often insufficient to meet these rigorous demands. The fundamental bottleneck arises from the extensive combinatorial complexity inherent in protein architecture, which manifests as a “needle in a haystack” problem where, for a protein of *N*_res_ residues, the conformational space scales at approximately 3^Nres^ (representing the three primary dihedral angles*φ, ψ, ω—*per residue), while the sequence space expands by 20^Nres^. Identifying an optimal solution, a sequence that not only reaches its thermodynamic global energy minimum but also fulfills a specific geometry for function, is computationally intractable through exhaustive enumeration. Furthermore, the high‐dimensional energy landscapes governed by van der Waals forces, hydrogen bonding, and hydrophobic effects often lead to misfolding or aggregation in repurposed natural proteins. Therefore, traditional methods remain tethered to the “evolutionary history” of known folds, limiting our ability to create entirely novel architectures tailored for synthetic immune circuits [15].

To address these limitations, de novo protein design offers a new approach, involving creating synthetic architectures predicated on thermodynamic laws, geometric constraints, and parametric equations, etc., yielding sequences that are distinct from the natural protein repertoire [16, 17]. Unlike traditional protein engineering, which modifies naturally occurring proteins, de novo design explores the full sequence space comprising 20^200^ possible combinations for a typical 200‐residue protein, a vast landscape from which evolution has sampled only an infinitesimal subset [16, 18]. This methodology operates on the hypothesis that proteins fold into the lowest energy states accessible to their sequences [19]. By utilizing physics‐based energy functions to model interatomic interactions such as van der Waals forces, electrostatics, hydrophobic effect, and hydrogen bonding, researchers can identify sequences that achieve a global energy minimum for a predefined target architecture [20]. This bottom‐up design strategy enables the creation of robust protein architectures with predictable atomic‐level features. In several pivotal studies, the resulting experimental structures have been shown to align closely with the underlying computational models [21, 22]. Furthermore, de novo design enables the engineering of bespoke functionalities that transcend the limitations of native proteins within the landscape of tumor immunotherapy. This includes the optimization of adoptive cell therapies by replacing unstable single‐chain variable fragment (scFv) domains with robust de novo binders (DNDBs), the development of selective cytokine mimics or receptor antagonists to fine‐tune immune responses, and the precise rewiring of intracellular signaling pathways to enhance the persistence and antitumor efficacy of engineered immune cells. By decoupling protein architecture from evolutionary constraints, de novo design provides a robust platform for developing the next generation of precision immunotherapeutics. Consequently, there is a compelling need for the field to actively embrace and explore these synthetic architectures to overcome the inherent limitations of natural proteins and try to fully unlock the untapped therapeutic potential of precision oncology.

**FIGURE 1:**
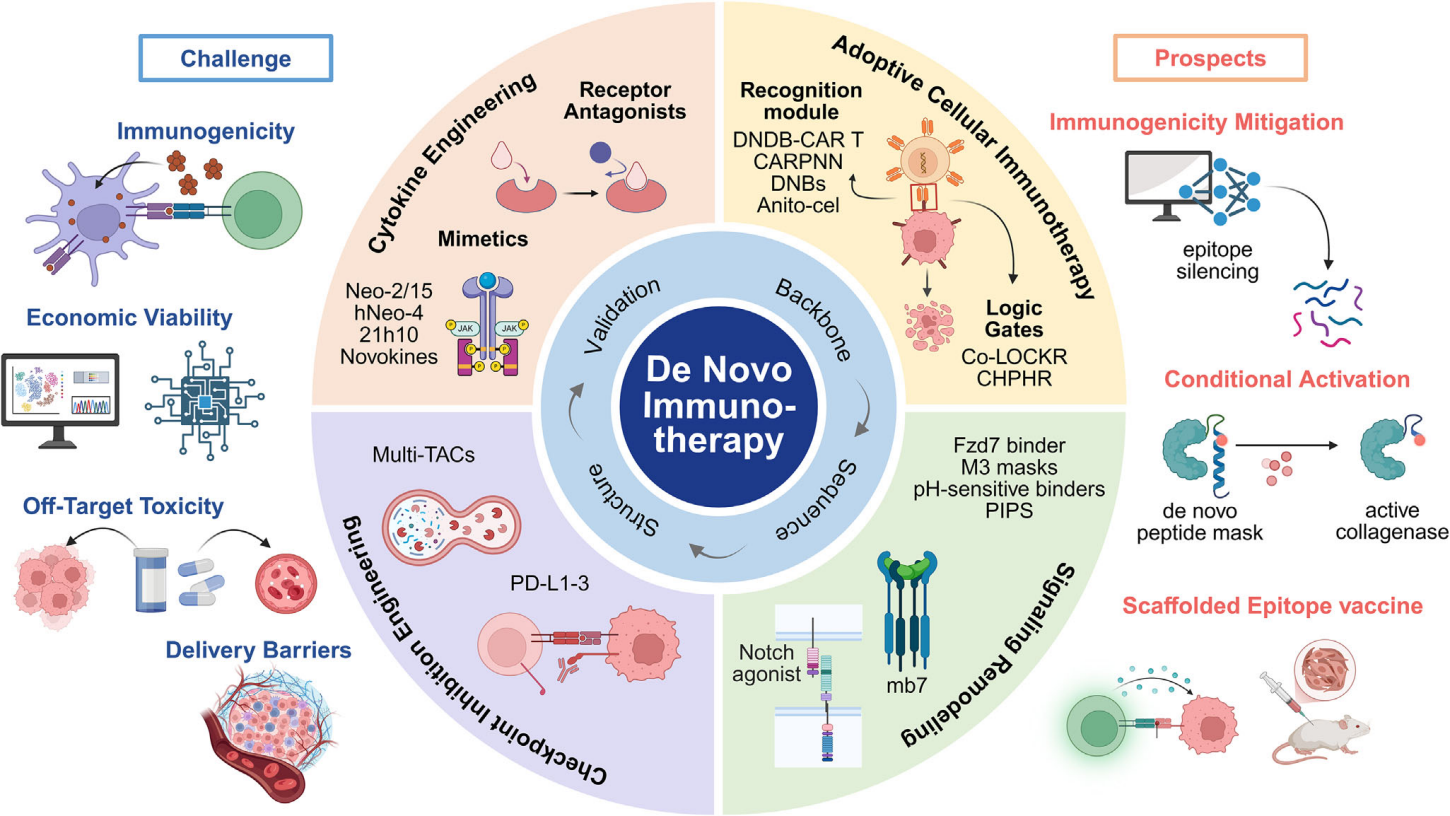
De novo protein design in cancer immunotherapy: current applications, limitations, and future directions. The central diagram illustrates the iterative computational workflow, where structural backbone definition and sequence design are integrated through a continuous cycle of structure prediction and validation. This foundational process drives four pivotal frontiers in cancer immunotherapy: cytokine engineering aimed at developing functional mimetics (e.g., Neo‐2/15, Novokines) and receptor antagonists; adoptive cellular immunotherapy featuring synthetic recognition modules (e.g., Anito‐cel, CARPNN) and complex logic gates (e.g., Co‐LOCKR, CHPHR) to enhance CAR‐T specificity; signaling remodeling utilizing tools such as Notch agonists, pH‐sensitive binders, and peptide masks to modulate intracellular pathways; and checkpoint inhibition engineering exemplified by precision binders like PD‐L1‐3 and Multi‐TACs. The framework also addresses the clinical landscape, highlighting major challenges such as immunogenicity, off‐target toxicity, delivery barriers, and economic viability, alongside forward‐looking prospects including immunogenicity mitigation via epitope silencing, conditional activation mechanisms, and the development of scaffolded epitope vaccines (figure was created with Biorender.com).

This review aims to explore the major applications, key breakthroughs, and future prospects of de novo protein design in cancer immunotherapy (Figure 1). This work first demonstrates a systematic computational workflow for de novo protein design. Subsequently, it highlights the broad potential of these synthetic proteins in cancer immunotherapy, focusing on four pivotal frontiers: cytokine engineering, adoptive cell therapy, signaling pathway remodeling, and immune checkpoint inhibition. Finally, the manuscript provides an analysis of the challenges and limitations inherent in clinical translation while offering a forward‐looking perspective on future directions and potential solutions.

## 2 De Novo Protein Design

**FIGURE 2:**
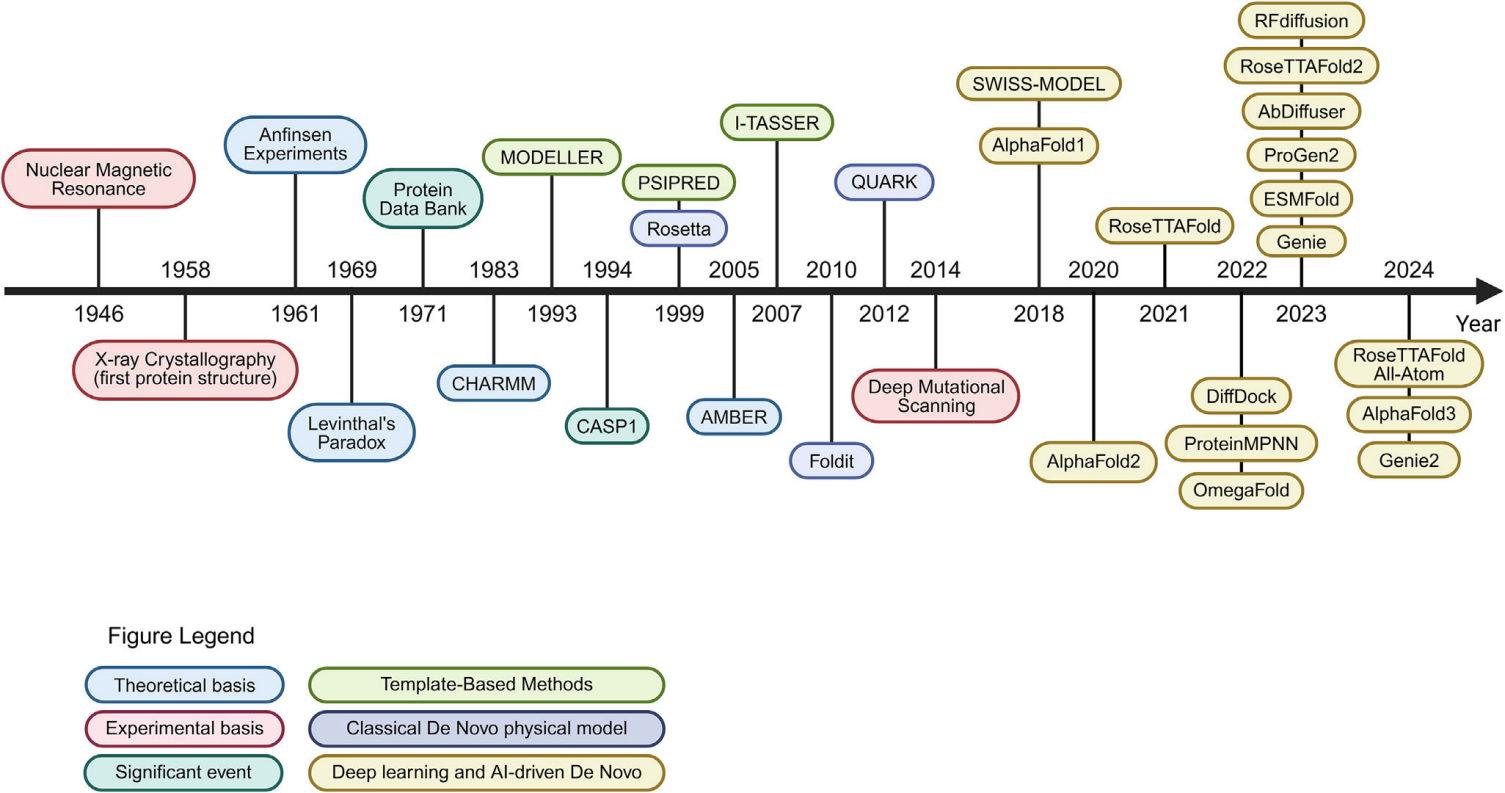
Timeline of protein structure modeling and de novo design. The evolutionary trajectory of protein structural biology and computational design, spanning from 1946 to 2024, reflects a profound shift from empirical observation to generative synthesis. This progression was anchored by the early synergy between experimental breakthroughs in X‐ray crystallography and Nuclear Magnetic Resonance (NMR) and the theoretical foundations laid by Anfinsen's Dogma, which established that a protein's primary sequence dictates its native structure, and Levinthal's Paradox, which underscored the immense computational challenge of the folding landscape. The establishment of the Protein Data Bank (PDB) in 1971 transformed these biological insights into a structured digital asset, enabling the rise of physics‐based force fields such as CHARMM and AMBER. These classical models leveraged Newtonian mechanics to simulate molecular dynamics and seek global energy minima, providing a rigorous but computationally intensive framework for understanding protein stability and motion. As structural data accumulated, the field transitioned toward statistical and template‐based methods that utilized evolutionary information. MODELLER and SWISS‐MODEL pioneered homology modeling by assuming that sequence similarity implies structural conservation, while the Rosetta software suite introduced a landmark approach to de novo design through fragment assembly and Monte Carlo sampling. This era sought to balance physical realism with statistical likelihood, a trend further refined by threading algorithms like I‐TASSER. However, the true paradigm shift arrived with the deep learning revolution, where tools like AlphaFold2 and RoseTTAFold leveraged Transformer architectures and co‐evolutionary signals from multiple sequence alignments (MSAs) to solve the protein‐folding problem with near‐experimental accuracy. This breakthrough fundamentally reoriented the field from predicting existing natural folds to the end‐to‐end mapping of sequence to 3D coordinates. The most recent frontier marks the transition into the generative AI era, where the focus has moved from understanding natural proteins to the bespoke creation of novel functional molecules. Diffusion‐based models, exemplified by RFdiffusion, enable the assembly of complex protein backbones from scratch, while ProteinMPNN facilitates the inverse folding problem by assigning stable, designable sequences to these artificial scaffolds. The culmination of this lineage is seen in multimodal systems such as AlphaFold3, which integrate proteins, nucleic acids, and small‐molecule ligands into a unified predictive framework. This historical arc demonstrates a move toward programming biology, where the integration of structural data, physical constraints, and deep generative architectures allows for the high‐precision design of enzymes, binders, and therapeutics that have never existed in nature (figure was created with Biorender.com).

The conceptual origins of de novo can be traced to the mid‐20th century, when Levinthal articulated his famous paradox, illuminating the fundamental complexities underlying protein folding mechanisms (Figure 2) [23]. The theoretical framework began to manifest experimentally in the 1980s through the pioneering work of William F. DeGrado, who demonstrated that stable protein architectures could indeed be constructed from synthetic α‐helical peptides [24]. A significant advancement emerged in 1997 when Dahiyat and Mayo introduced the first automated computational paradigm, employing energy‐based algorithms to systematically select amino acid sequences that would fold into predefined three‐dimensional structures [25]. Building upon this foundation over the following decade, computing power gave rise to sophisticated tools like Baker Lab's Rosetta, which by the early 2000s enabled the design of highly stable proteins with near‐atomic precision [26, 27]. Contemporary de novo protein design has evolved into an established discipline, finding applications across diverse fields including medicine, materials science, and synthetic biology.

### 2.1 Core Principles

Unlike rational design or directed evolution, de novo protein design aims to create proteins from scratch to achieve specific biological functions, independent of any predefined natural templates [21]. This approach emphasizes structure‐function innovation, enabling the design of novel proteins with precisely engineered binding interfaces and superior thermodynamic stability. However, without a natural blueprint to guide the folding process, the primary challenge lies in identifying which of the nearly infinite possible amino acid sequences will adopt a stable, functional conformation. This necessitates a robust energy function—a computational scoring system that serves as the ultimate criterion of structural viability [26, 28]. By accurately capturing the spatial relationships and interatomic forces within a protein, these physics‐based energy functions, which are rooted in the first principles of molecular motion, allow researchers to identify the native or most stable state through energy minimization [26]. Ultimately, these sophisticated frameworks provide the atomic‐resolution precision required to describe the precise coordination of nonbonded atom‐pair interactions, backbone and side‐chain torsional preferences, and nonideal bond lengths and angles, ensuring that designed molecules are physically achievable.

### 2.2 Computational Strategies

**FIGURE 3:**
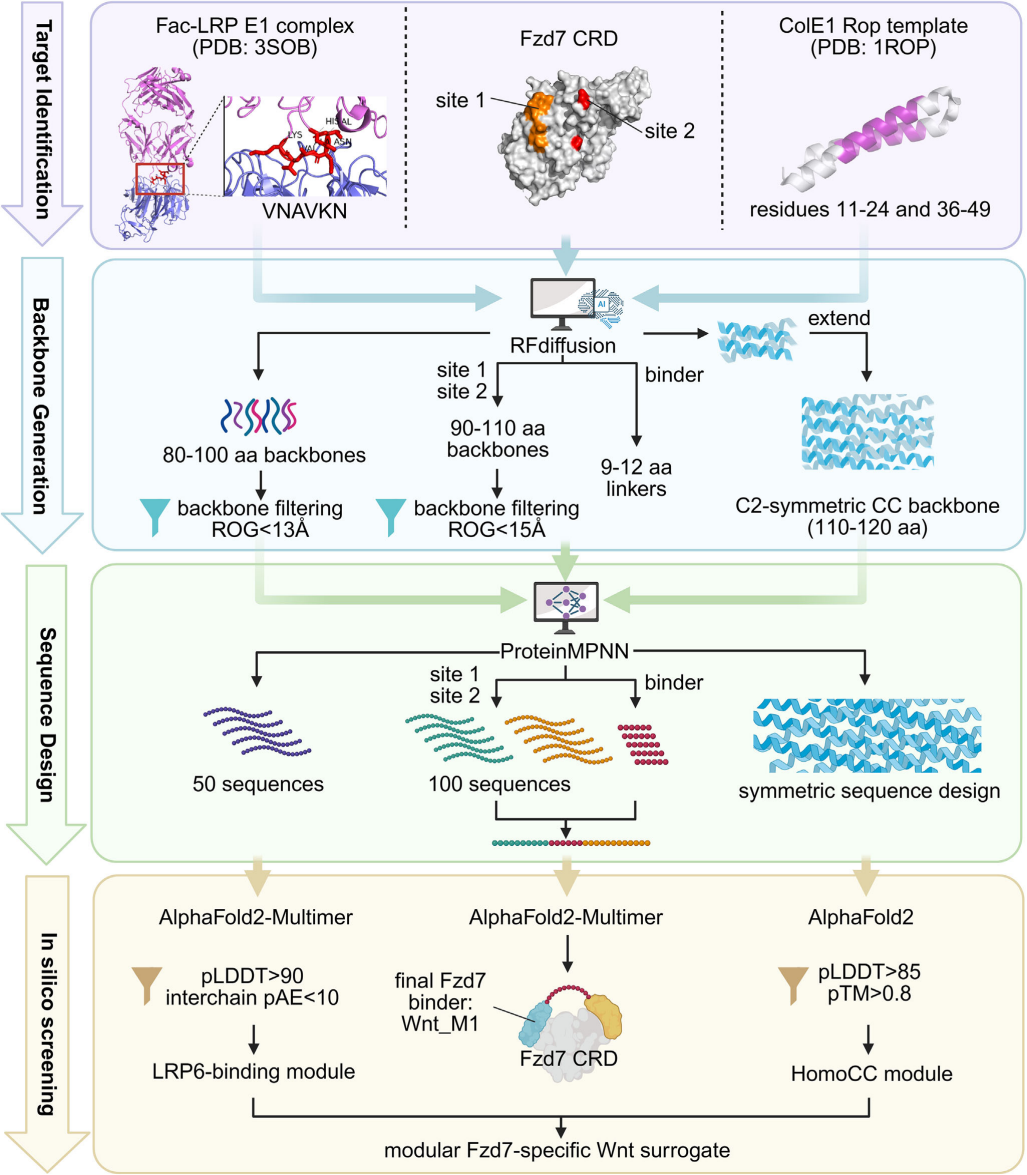
A representative de novo design pipeline for a Fzd7‐specific modular Wnt surrogate (adapted from the work of Junyup Lee, Byung‐Ha Oh, et al. [29]). This figure delineates a comprehensive computational workflow for designing functional synthetic proteins, using a Fzd7‐specific Wnt surrogate as a representative case. The process integrates generative AI, physics‐based filtering, and deep learning‐based validation to assemble modular functional units. Target identification and structural analysis. The design process was initiated with the structural characterization of three key functional modules. For the LRP6‐binding module, the VNAVKN epitope was extracted from the Fab–LRP6 E1 complex (PDB: 3SOB). In the absence of an experimental co‐crystal structure for the Fzd7 CRD–Wnt complex, the human Fzd7 CRD structure was computationally predicted using AlphaFold‐Multimer (AF‐Multi) as the primary design target. The crystal structure of the Xenopus Wnt8–mouse Fzd8 CRD complex (PDB: 4F08) was employed strictly as a representative schematic to illustrate the spatial orientation of the palmitoleic acid (PAM) moiety and to delineate the two conserved hydrophobic interaction surfaces on the Fzd7 CRD: Site 1 (adjacent to the lipid‐binding groove; hotspots Leu81, Pro88, Phe138) and Site 2 (the Wnt “index‐finger” interaction surface; hotspots Phe100, Val109, Val161). The dimerization scaffold was derived from the antiparallel coiled‐coil motif of the ColE1 Rop protein (PDB: 1ROP). Backbone generation via RFdiffusion. Scaffold backbones were generated using RFdiffusion. LRP6‐binding backbones (80–100 residues) were filtered for compactness (Radius of Gyration, ROG < 13 Å). Fzd7‐binding modules were designed in two stages: primary backbones (90–110 residues) targeting Site 1, and secondary linkers (9–12 residues) targeting Site 2 (ROG < 15 Å). The homodimeric coiled‐coil (HomoCC) backbones were generated with C_2 symmetry, resulting in 110–120 residue antiparallel dimers. Sequence design and machine learning optimization. ProteinMPNN was employed to optimize sequences for the generated backbones. A library of 50 sequences per backbone was generated for the LRP6 module, while 100 sequences per backbone were designed for the Fzd7 modules to ensure high‐affinity binding. Symmetric sequence design was applied to the HomoCC module to maintain structural stability and dimeric orientation. In silico screening and modular assembly. Candidate designs were validated using AlphaFold2 and AlphaFold2‐Multimer. Selection criteria included high confidence scores (pLDDT > 90 for LRP6/Fzd7 modules; pLDDT > 85 for HomoCC), low interchain predicted Alignment Error (predicted aligned error < 10), and high predicted TM‐score (pTM > 0.8). The lead Fzd7‐binder (Wnt_M1) was integrated with the LRP6‐binding module and the HomoCC scaffold to form the final modular Fzd7‐specific Wnt surrogate, designed to bridge Fzd7 and LRP6 to mimic canonical Wnt signaling (figure was created with Biorender.com).

Through the synergistic power of deep learning networks, sophisticated sequence optimization algorithms, and high‐resolution molecular dynamics simulations, scientists now wield enhanced capability to design proteins from digital blueprints into experimentally validated molecular machines. This section outlines the four primary stages of the de novo protein design pipeline, highlighting the methodological shift from classical stochastic sampling to deep‐learning‐driven generation, while also discussing the essential role of physics‐based refinement and dynamic validation (Figure 3).

#### 2.2.1 Defining Target Structures and Generating Backbones

The de novo design process commences with the crucial first step: generating reasonable protein backbone structures through computer modeling, based on geometric and physical principles, to achieve desired functionalities. Historically, this phase relied on stochastic conformational searching via Monte Carlo sampling (1940s), which navigated the vast energy landscape based on geometric and physical principles to identify low‐energy states. The rapid advancement of artificial intelligence has fundamentally streamlined this stage through generative approaches. RFdiffusion [30] represents a methodological improvement by employing diffusion models and generative denoising algorithms to sculpt complex protein topologies from noise. However, RFdiffusion primarily focuses on backbone topology, typically necessitating subsequent, decoupled steps for sequence design and side‐chain placement. Diverging from this backbone‐first paradigm, BoltzGen [31] represents a shift toward all‐atom unified modeling. By unifying protein design and structure prediction within a single generative framework, BoltzGen simultaneously optimizes main‐chain coordinates and side‐chain conformations. This integration allows for superior structural reasoning regarding target‐binder interactions, enabling the precise design of cross‐modal binders—such as nanobodies or peptides—against diverse targets, including small molecules and disordered proteins [31]. In summary, while the various methodologies discussed here are capable of precisely defining three‐dimensional geometric templates for proteins, these abstract coordinates must be assigned a specific chemical identity to attain physical stability. This logical shift from structural definition to amino acid sequence searching constitutes the inverse folding problem, the process of identifying sequences for a target structure [21, 32, 33].

#### 2.2.2 Sequence Design for Target Backbones

With a target backbone defined, the next crucial step is to determine an appropriate amino acid sequence that will adopt this specific fold while ensuring stability and conferring the intended function. This involves decorating the backbone.

Classical computational tools laid the groundwork; RosettaDesign [21], using physics‐based energy functions and Monte Carlo simulated annealing, optimized sequences for low‐energy states and features like stable hydrophobic cores. EvoDesign incorporated evolutionary insights by leveraging structural profiles and sequence‐structure compatibility scores derived from homologous proteins in the PDB. Instead of relying solely on force field calculations, it selects amino acids based on conserved patterns observed in nature, ensuring that the designed sequences align with established evolutionary design rules [34]. Building on these advancements, ProteinMPNN (message passing neural network) represents a paradigm shift toward deep‐learning‐driven sequence design, utilizing a message passing neural network to extract geometric features—including interatomic distances, frame orientations, and dihedral angles—from protein backbones. By extracting geometric features—such as interatomic distances and dihedral angles—from protein backbones, the model effectively maps these 3D coordinates to amino acid sequences with superior probabilistic accuracy and computational efficiency, demonstrating exceptional robustness to backbone inaccuracies [35].

To conclude, beyond the aforementioned foundational thermodynamic principles, the primary objectives of sequence design focus on computational implementation and fold specificity. This involves optimizing the amino acid sequence to reach a global energy minimum for the target scaffold through dense hydrophobic core packing and the engineering of precise, high‐affinity interaction interfaces.

#### 2.2.3 Structure Prediction and Refinement

After designing a sequence for a backbone, it is essential to predict the actual three‐dimensional structure that this specific sequence is most likely to adopt and to computationally refine this structure. AI‐driven protein structure prediction models have become critical here.

AlphaFold2, developed by DeepMind in 2021, has established itself as a leading platform for high‐precision prediction, achieving average TM‐scores of 0.88 on CAMEO (Continuous Automated Model EvaluatiOn) and 0.85 on CASP14 (Critical Assessment of Techniques for Protein Structure Prediction) by leveraging self‐attention mechanisms, algorithms that allow the model to dynamically weight the importance of different amino acid residues to capture complex, long‐range spatial dependencies, to achieve near‐experimental precision in protein structure prediction [36, 37]. RoseTTAFold offers a resource‐efficient alternative by employing a three‐track neural network that simultaneously processes 1D sequence information, 2D distance maps, and 3D atomic coordinates [15]. This tri‐track architecture allows information to flow bidirectionally between different levels of structural representation, enabling the model to excel in protein complex modeling, though typically with slightly lower accuracy than AlphaFold2 in certain monomeric cases, as shown in Table 1 [15]. ESMFold represents a methodological shift and advancement in computational structural biology by leveraging the sophisticated evolutionary scale modeling (ESM) framework to enable rapid, single‐sequence protein‐folding predictions relying on statistical learning of the evolutionary sequence in nature. This transformer‐based architecture demonstrates remarkable computational efficiency, executing folding predictions up to 60‐fold faster than AlphaFold2 while maintaining competitive accuracy metrics, thereby establishing it as an exceptionally well‐suited tool for high‐throughput de novo protein design pipelines, large‐scale virtual screening campaigns, and iterative design‐build‐test‐learn cycles that demand rapid structural assessment of vast combinatorial libraries of engineered protein variants. ESMFold extracts structural information directly from protein language model (PLM) embeddings, eliminating the need for computationally intensive multiple sequence alignment (MSA). By bypassing traditional database searches and memory‐heavy preprocessing, it enables faster and more scalable structure prediction [38]. Beyond initial prediction, further computational refinement is often necessary. A primary tool for this is RosettaRelax, an algorithm developed by the Baker group that performs iterative cycles of side‐chain repacking and small‐scale backbone torsion movements. By navigating the local energy landscape, RosettaRelax resolves steric clashes and optimizes hydrogen‐bonding networks. This process is often complemented by energy minimization using classical molecular mechanics force fields, such as AMBER or CHARMM, which refine local geometries and interatomic interactions to ensure the design sits at a robust, low‐energy conformation.

**TABLE 1:** Comparison of protein structure prediction models.

Model	Applications	Key principles and design	Accuracy (TM‐score)	Advantages	Limitations
AlphaFold	High‐accuracy prediction of single protein structures Benchmark for CASP13 [39]	Utilizes Convolutional Neural Network (CNN) to extract evolutionary features from MSA [36, 39]. Incorporates physical and geometric constraints		Potential for RNA–protein interaction predictions	High computational demands. Limited accuracy for protein complexes and dynamic systems
AlphaFold2	Accurate prediction of complex protein structures. Reference for experimental validation	Improved transformer network with attention mechanism for long‐range interactions. End‐to‐end training integrating sequence and geometrical constraints	0.88 (CAMEO) No MSA:0.41 (CAMEO) 0.85 (CASP14) No MSA:0.38 (CASP14)	Exceptional accuracy for single proteins and some complexes [40]. Strong generalization capabilities	Requires significant computational resources. Limited for RNA‐protein complexes
RoseTTAFold	De novo protein design. Rapid, efficient structure prediction	Three‐track network: Sequence track (amino acid features), MSA track (evolutionary features), and geometry track (distance/angle constraints) [15]	0.82 (Standard CAMEO) 0.47 (No MSA, CAMEO) 0.81 (Standard, CASP14) 0.37 (No MSA, CASP14)	Computationally efficient. Produces high‐quality predictions in reasonable time	Slightly lower accuracy than AlphaFold2. Limited generalization for specific protein families
ESMFold	Single‐sequence structure prediction. High‐throughput protein screening	ESM using transformer architecture. Does not rely on MSA, enabling rapid folding predictions [38]	0.83 (CAMEO) Standard 0.68 (CASP14) Standard	6 times faster than AlphaFold2 (a single NVIDIA V100 GPU, a 384‐amino‐acids protein). Ideal for large‐scale protein libraries	Lower accuracy for complex structures. Not suitable for high‐precision applications

However, a low‐energy static model does not guarantee functional success; experimentally viable de novo proteins must exhibit structural integrity and thermodynamic stability under fluctuating physiological conditions [41]. To bridge this gap, molecular dynamics (MD) simulations, using packages like GROMACS and NAMD, are employed to transition from static snapshots to time‐dependent behavior [42, 43]. By simulating the protein within a biologically relevant environment, including explicit solvent molecules and ionic strength, MD provides critical insights into structural flexibility, solvent‐exposed surface stability, and mechanical robustness. In summary, the integration of computational refinement and dynamic simulations provides a rigorous validation framework, ensuring that designed candidates possess the requisite thermodynamic stability and conformational resilience before transitioning to costly experimental synthesis.

### 2.3 Experimental Validation for Structural and Biophysical Characterization

Given that de novo proteins are engineered from first principles without evolutionary templates, rigorous experimental validation is indispensable to bridge the gap between computational prediction and biological reality.

Common validation methods include X‐ray crystallography, cryo‐electron microscopy (Cryo‐EM), circular dichroism (CD), surface plasmon resonance (SPR), and isothermal titration calorimetry (ITC). It is critical to note that these techniques address distinct evaluation metrics: X‐ray crystallography can precisely define the three‐dimensional structure of proteins, evaluating folding accuracy to ensure that the designed combination interface achieves geometric complementarity with the expected immune target; Cryo‐EM is ideal for larger or difficult‐to‐crystallize proteins, helping to elucidate their functional conformations [44]. Furthermore, thermodynamic stability parameters, including melting temperature (Tm) and free energy of unfolding (ΔG unfolding), are rigorously determined through CD‐based thermal denaturation or differential scanning fluorimetry (DSF) [45]. Diverse biological functionalities ranging from enzymatic activity and substrate specificity to allosteric regulation are validated via specific biochemical assays. Binding affinity and kinetic parameters (kon, koff, KD) are precisely quantified by SPR and ITC [46, 47]. For novel immunotherapy agents such as immune checkpoint inhibitors or cytokine mimetics, the subpicomolar (sub‐pM) affinity confirmed through these techniques provides a critical foundation for predicting clinical dosages, achieving precise targeting, and mitigating off‐target toxicities. Ultimately, the combined use of these validation methods and metrics provides a target‐oriented evaluation of fitness‐for‐purpose, ensuring that the designed protein satisfies its specific functional requirements and overcomes the biological barriers it was engineered to address. While structural and biophysical characterization confirms the physicochemical fidelity of de novo designs, cell‐based and in vivo evaluations are not discussed in this section, as their specific methodologies are intrinsically tied to the unique biological pathways and therapeutic goals of each protein.

## 3 Applications of De Novo Design

The applications of de novo design in cancer immunotherapy mainly include cytokine modulation, adoptive cell therapy, remodeling of signaling pathways, and immune checkpoint inhibitors (Table 2).

**TABLE 2:** Representative applications of de novo protein design in cancer immunotherapy.

Category	Subfield	Representative molecule(s)	Core advantages	References
Cytokine engineering	Cytokine mimetics	Neo‐2/15	Eliminates IL‐2Rα (CD25) binding to avoid Treg activation and vascular leak syndrome	[48, 49]
		21h10 (IL‐21 mimic)	Extends pSTAT3 signaling duration from hours to >24 h	[50]
		Novokines (library of >1000 agonists)	Enables biased signaling by modulating receptor subunit geometry	[51]
	Receptor antagonists	IL‐6R/IL‐1R1 antagonists	Effectively blocks signaling pathways involved in CRS	[52]
		IL‐23R minibinders	Picomolar (pM) affinity; hyperstable and resistant to proteolysis, enabling successful oral delivery in colitis models	[53]
Adoptive cellular therapy	scFv replacement	Anitocel (D‐domain)	Reduced tonic signaling, 97%–100% ORR in RRMM patients (iMMagine‐1 trial) and high deep‐response rates (CR ≥79% and 68% respectively; 89% MRD negativity)	[54, 55, 56, 57]
		BIKES	NY1‐B04 BIKE‐T cells induced rapid cell death in the A375 melanoma cell line within 24 h	[58]
		DNBs (for BCMA)	Overcomes teclistamab resistance caused by the BCMA‐R27P mutation	[59]
	Logic gates	Co‐LOCKR	Prevents on‐target off‐tumor toxicity	[60]
		CIPHR	Downregulates TIM3 to mitigate T‐cell exhaustion	[61]
Signaling pathway remodeling	Valency and geometry	mb7 (FGFRc binder)	Proves that FGFR activation depends strictly on receptor valency and spacing	[62]
	Molecular switches	PIPS	Kinase‐responsive toggle	[63]
		Peptide masks	Provides >1000‐fold affinity modulation upon protease cleavage	[64]
	Subtype selectivity	Wnt surrogate (Fzd7)	Specific for Fzd7 (Kd < 23 nM); Aims to turn cold tumors hot by blocking subtype‐specific Wnt signaling	[29]
	Integrin inhibition	B6_BP_dslf	Selective inhibitors for αvβ6/αvβ8; picomolar binding affinity to reduce fibrotic/tumor burden	[65]
Immune checkpoints	Geometric scaffolds	Concave scaffolds	Specific for convex topological structures such as CTLA‐4 and PD‐L1, geometrically complementary concave binders are designed to enhance the affinity	[66]
	Noninvasive imaging	PD‐L1‐3	Submolar (<1 pM) affinity; the imaging contrast is high, and the imaging can be performed 5 to 30 min after injection	[67]
	Protein degradation	Multi‐TACs (GG56)	Tumor‐specific degradation of PD‐L1 via adsorption‐mediated endocytosis Significant extension of median survival from 9 days to 35 days in melanoma models	[68]

### 3.1 Cytokine Engineering via De Novo Design

As central orchestrators of the immune system, cytokines represent a pivotal strategy to overcome tumor immune evasion by robustly activating effector immune cells and remodeling TME [69, 70]. The primary therapeutic utility of cytokines encompasses using agonists to replace deficient factors or amplify immunity in cancer and infection, as well as employing antagonists to suppress pathological inflammation and autoimmune disease, and so on [71, 72, 73]. Despite their therapeutic potential, natural cytokines are characterized by pleiotropy, where they mediate more than one action across diverse cell types, and redundancy, which complicates single‐pathway targeting [69, 74]. Furthermore, their clinical efficacy is often compromised by unfavorable physicochemical and pharmacokinetic properties, such as high thermodynamic instability and rapid renal clearance, leading to a short circulating half‐life that necessitates high‐dose administration and triggers severe systemic toxicities [75, 76]. To counteract the biological challenges of cytokine pleiotropy and inadequate in vivo stability, researchers are utilizing de novo computational protein design to construct cytokine mimetics alongside engineered receptor antagonists.

#### 3.1.1 Cytokine Mimetics

Given the exponential increase in clinical research, a wide array of cytokine mimetics and engineered formulations are currently under evaluation, among which Interleukin‐2 (IL‐2) represents the most extensively studied and paradigmatic candidate [77, 78]. However, the clinical utility of IL‐2 is fundamentally restricted by its severe systemic toxicity and its inherent capacity to activate immunosuppressive regulatory T cells (Tregs) through binding with the high‐affinity IL‐2Rα (CD25) subunit [79]. To address these structural and pharmacological limitations, researchers utilized parametric equations to idealize the four core α‐helices (H1‐H4) of IL‐2. Combined with the Rosetta platform, they executed backbone sequence design and interface optimization to construct a 100‐amino acid protein. This protein possesses a topology distinct from the natural cytokine, with a sequence identity of approximately 14%. Structurally, Neo‐2/15 eliminates the binding interface with IL‐2Rα (CD25) while maintaining affinity for the IL‐2Rβγ chain heterodimeric receptor. In terms of biological effects, the molecule activates effector T cells while avoiding immune suppression mediated by regulatory T cells (Treg) and vascular leak syndrome [48, 80, 81, 82]. Besides, other researchers also found out that by augmenting MHC‐I expression and diversifying the intratumoral T‐cell receptor repertoire, the Neo‐2/15 drives potent antitumor efficacy both as a monotherapy and in synergistic combination with PD‐1 inhibition [49]. Furthermore, to mitigate systemic hyperactivation resulting from receptor engagement in nontumor regions, the David Baker team developed two‐component split versions of Neo‐2/15. Upon evaluating multiple splitting strategies, the combination of the H1 and H32’4 fragments was identified as the optimal pair. Biochemically, these two split fragments exhibit low intrinsic affinity for one another in the absence of IL‐2 receptor subunits (dissociation constant [*K*_d_] ∼4.5 µM), but in the presence of soluble IL‐2Rβ and IL‐2Rγ, they assemble into a complex with significantly enhanced affinity (*K*_d_ = 50.8 nM). This property is highly desirable, as it ensures that the functional reconstitution of the cytokine mimetic occurs specifically at the intended target site through receptor‐mediated stabilization [83].

Similarly, based on the modular scaffold of Neo2, researchers engineered a receptor‐exclusive mimetic of IL‐4. As a defining cytokine of type 2 immunity, IL‐4 acts as a critical orchestrator of both host‐protective functions, ranging from antihelminth defense and metabolic homeostasis to tissue repair, while simultaneously driving pathogenic processes, including the induction of allergic inflammation and fibrotic disorders when dysregulated [84, 85]. However, native IL‐4 exhibits pleiotropic actions by signaling through both the type I receptor complex (IL‐4Rα and γc), primarily expressed on hematopoietic cells, and the type II complex (IL‐4Rα and IL‐13Rα1), which can drive unintended inflammatory responses [86, 87, 88]. To address this, 14 key amino acid residues from the hIL‐4/hIL‐4Rα interface were identified, and the ternary structure of Neo‐2 in complex with hIL‐2Rβ and γc was then computationally aligned with the native hIL‐4/hIL‐4Rα/γc complex. These residues were subsequently grafted onto the Neo‐2 scaffold. The resulting construct was subjected to random mutagenesis and four rounds of selection using a yeast surface display platform to enhance its binding affinity for hIL‐4Rα. This process culminated in the identification of hNeo‐4, which incorporated a total of 16 mutations and exhibited potent, highly selective receptor binding [89].

The redesign of IL‐21 focused on kinetic stability. IL‐21, as a member of the same common gamma chain receptor family as IL‐2 and IL‐15, is a pleiotropic cytokine primarily secreted by activated CD4^+^ T cells that orchestrates potent immune responses by activating the STAT3 signaling pathway, which enhances the cytotoxic activity of CD8^+^ T cells and NK cells while promoting B cell differentiation into antibody‐secreting plasma cells [90]. The native IL‐21 cytokine adopts an “up–up–down–down” topology, a configuration that necessitates two long, unstructured loops on its backside to connect the similarly oriented helices. These disordered regions significantly reduce the molecule's overall stability and render it highly susceptible to protease‐mediated degradation, thereby limiting its clinical utility. To address these structural vulnerabilities, the 21h10 mimetic was engineered by implementing a de novo “up–down–up–down” four‐helix bundle topology. This architectural shift, which alternates the orientation of adjacent helices, allows for more ideal helical packing and the use of significantly shorter, well‐ordered connecting loops. The design process involved the parametric sampling of helical geometries within Rosetta to establish an idealized four‐helix bundle that preserves the essential receptor‐binding interfaces for IL‐21R and γ chain. By eliminating the poorly ordered segments inherent in the natural cytokine, this structural restructuring significantly enhances the protein's kinetic stability and extends the duration of induced pSTAT3 signaling from several hours to over 24 h. This prolonged signaling promotes robust effector CD8^+^ T cell differentiation and enhances antitumor activity across a broad range of TCR affinities [50].

Beyond the targeted de novo cytokine engineering mentioned above, researchers have developed a versatile de novo protein design platform for generating diverse cytokine mimetics from the target receptor structure alone. By systematically modulating receptor geometry, the study successfully decoupled the competitive functions of various cytokines and yielded diverse Novokines. These include Novo7 (an IL‐7 mimetic), NovoIFNα (a Type I interferon mimetic), and Novo10 (an IL‐10 memetic), each of which was engineered to achieve biased signaling by stabilizing specific receptor conformations. This work establishes receptor geometry as a central determinant of cytokine activity, showing it to be as critical as receptor identity or binding affinity in orchestrating specific immune outcomes [51].

Similarly, by engineering nonnatural receptor pairings, researchers can elicit signaling outcomes that surpass the functional limits of natural cytokines, such as redirecting T cells toward monocyte‐like fates or inducing proliferation profiles—including selective CD4^+^ T‐cell expansion—that are absent in their natural counterparts. This approach allows for the reprogramming of cellular responses by combining signaling subunits from different families, thereby enabling functions like driving monocyte proliferation or enhancing T‐cell survival through noncanonical receptor clusters. Researchers employed a high‐throughput de novo design approach centered on the computational generation and fusion of paired binding domains. By combining 33 distinct computationally designed receptor‐binding domains, comprising 9 newly designed binders targeting essential cytokine subunits (including γcommon, IL‐4Rα, and IFNAR1/2) and 24 previously developed domains targeting diverse classes, such as TNF receptors (OX40, 4‐1BB), immune checkpoints (PD‐L1, CTLA‐4), and growth factor receptors (EGFR, TrkA, HER2), a library of over 1000 potential Novokines″ was synthesized. Experimental validation demonstrated that 75 of these candidates successfully activated pSTAT signaling in peripheral blood mononuclear cells. Among these, IL‐21 and IL‐10 mimics exhibited broadened signaling outputs, such as the activation of pSTAT5 alongside pSTAT3, which translated into enhanced cellular functions like supporting CD4^+^ T cell proliferation at levels comparable to IL‐2—a capacity notably absent in their natural counterparts. Furthermore, the framework enabled the creation of Multikines, such as a hybrid IL‐2/IL‐21 agonist that activated both pSTAT5 and pSTAT3 pathways, thereby promoting T cell survival and resistance to exhaustion. By mapping these signaling activities across the entire receptor‐pairing matrix, the researchers identified an unexpected functional versatility in certain subunits. Crucially, the study discovered that the natural subunit IFNAR1 can function as a versatile common co‐receptor when paired with noncognate partners, analogous to the roles played by the natural γcommon or βcommon subunits [91].

In summary, the successful development of these de novo cytokine mimetics underscores a paradigm shift from modifying natural scaffolds to engineering structurally idealized agonists from first principles. By decoupling signaling activity from evolutionary constraints, this approach not only overcomes the inherent thermodynamic instabilities of native cytokines but also enables the highly specific activation of immune pathways, thereby significantly mitigating systemic toxicities.

#### 3.1.2 Cytokine Receptor Antagonists

Beyond direct cytokine mimetics, de novo technology has been applied to design high‐performance antagonists. Cytokine release syndrome (CRS) is an acute systemic inflammatory syndrome characterized by the pathological overproduction of potent immune regulators such as IL‐6 and IL‐1β, whose excessive release triggers severe tissue damage and organ injury [92, 93]. To address the need for more stable and precise safety switches, Buwei Huang et al. [52] utilized a Rosetta‐based protein design method to design protein antagonists targeting IL‐6R, Glycoprotein130, and IL‐1R1. They first selected hydrophobic residues overlapping with the cytokine binding sites and generated a set of rotamer interaction fields (RIF) around these residues. Then, using the Patchdock software, they rigidly docked a predesigned protein scaffold library with the target residues and performed local searches through the Rifdock protocol to optimize the side‐chain–side‐chain interactions. The results demonstrated that the designed antagonist effectively blocked IL‐1 and IL‐6 signaling pathways [52], demonstrating that de novo computational methodology can provide a robust and tunable framework for the clinical management of treatment‐induced hyperinflammation.

While Huang et al. focused on acute systemic safety, de novo technology also offers preferable solutions for remodeling the chronic, protumorigenic inflammatory microenvironment. Targeting Th17, IL‐23 primarily serves as an upstream regulator that induces and maintains Th17 cell stability, whereas IL‐17 acts as a core pro‐inflammatory effector that mediates host defense against pathogens while simultaneously driving the chronic inflammatory responses characteristic of various autoimmune diseases [94]. To address the inability of conventional antibodies to survive localized delivery or penetrate dense tissues, researchers de novo designed IL‐17A and IL‐23R minibinders that achieve picomolar affinities despite their minimal molecular size. This work specifically solves the challenge of biochemical vulnerability: these scaffolds exhibit exceptional resistance to heat, acidity, and proteolysis, maintaining structural integrity even within the gastrointestinal tract [88]. In the context of cancer immunotherapy, this biochemical robustness enables a potent, localized treatment strategy, such as oral administration for colorectal cancers, that directly neutralizes the protumorigenic milieu while circumventing the systemic toxicities and delivery barriers of traditional biologics.

In addition to neutralizing extracellular proinflammatory cytokines, de novo design technology can further penetrate the lipid bilayer to directly disrupt signaling mediators that drive tumor survival and proliferation. The erythropoietin receptor (EpoR) is a prototypical cytokine receptor where the transmembrane (TM) domain contributes to receptor homodimerization and the subsequent activation of the JAK‐STAT pathway, which facilitates malignant progression [95, 96]. To address the challenge of targeting TM regions that are deeply buried within the bilayer and inaccessible to conventional antibodies or small molecules, Mravic et al. [97] developed a workflow for the de novo design of TM proteins. Moving beyond the previous limitations of simply mimicking natural TM motifs, they computationally engineered a custom antiparallel topology designed to bind the EpoR TM domain. By specifically targeting the EpoR TM surface at a 1:1 stoichiometry, these synthetic computed helical antimembrane proteins (CHAMP) outcompete the native parallel homodimerization required for receptor signaling. This approach successfully inhibited EPO‐induced cell proliferation and silenced cross‐membrane signaling, providing a powerful tool to study and perturb bioactive TM regions [97].

Expanding from spatially restricted transmembrane regions, Maksymenko et al. [98] established the HECTOR dock‐and‐design platform, a generalizable computational pipeline that rapidly generates site‐specific binders for diverse oncogenic epitopes without extensive screening. Within this framework, researchers engineered anti‐VEGF (vascular endothelial growth factor) binders such as Sam0.7 (Tm = 63.1°C), which matched bevacizumab's in vivo tumor suppression at a significantly lower concentration (3.5 vs. 25 mg/mL), alongside IL‐7Rα antagonists like des03 that achieved 1.4 nM affinity and exceptional thermostability (Tm > 100°C) By demonstrating the capacity to target previously inaccessible epitopes, the HECTOR pipeline provides a generalizable platform for the on‐demand design of site‐specific binders for diverse oncogenic surfaces [98].

### 3.2 Adoptive Cellular Immunotherapy

Adoptive cell therapy (ACT), specifically chimeric antigen receptor (CAR) T‐cell therapy, represents a landmark milestone in cancer immunotherapy, achieving substantial clinical efficacy in hematological malignancies [99, 100, 101, 102, 103]. However, its broader clinical translation is still hindered by critical bottlenecks, such as the aforementioned CRS, the biophysical instability of natural scFv templates that triggers ligand‐independent tonic signaling, off‐target toxicities due to a lack of programmable specificity, and reliance on the density of surface‐antigen [104, 105, 106, 107, 108].

De novo protein design addresses these limitations by bypassing natural templates to engineer optimized modules, ranging from hyperstable binders and synthetic logic gates to pMHC‐targeting domains and custom‐topology transmembrane modulators. This approach ensures the precision, safety, and functional reach of ACT with atomic accuracy, effectively turning previously undruggable signaling hubs into accessible therapeutic targets.

#### 3.2.1 De Novo‐Designed Recognition Modules for Receptor Optimization

The extracellular antigen recognition domain of the CAR is typically composed of a scFv, designed to specifically recognize target tumor antigens, such as CD19 [109, 110]. However, scFvs often suffer from poor folding stability, which may interfere with antigen recognition efficiency and therapeutic outcomes, posing a challenge for clinical applications [107, 111]. To circumvent these structural vulnerabilities, de novo protein design offers a robust alternative by creating hyperstable, minimal binders that bypass the limitations of natural antibody templates. As a compelling example, to target the overexpressed surface antigens EGFR and CD276 in glioblastoma (GBM), the research team developed DNDB‐CAR T technology, utilizing DNDBs to replace traditional scFvs, which are prone to aggregation and structural instability. Specifically, by employing computational methodologies such as RifGen/RifDock in conjunction with yeast surface display screening, the researchers engineered antigen‐binding interfaces characterized by atomic accuracy, high biophysical stability, and picomolar affinity against EGFR and CD276. Experimental validation demonstrated that DNDB‐CAR T cells significantly improved receptor surface expression and cytotoxic potency, while exhibiting enhanced proliferative capacity and superior resistance to exhaustion de novo. Chow et al. also developed a framework to replace scFvs with de novo protein binders, screening 1589 candidates against BCMA, CD19, and CD22. They established the CARPNN (chimeric antigen receptor–protein–MPNN) workflow to diversify noninterface residues via ProteinMPNN, demonstrating that tuning parameters like net charge effectively eliminate tonic signaling and off‐target liabilities while maintaining high killing specificity. This process identified optimized binders B5.10 (targeting BCMA) and D1.N0 (targeting CD22) with performance matching or exceeding that of clinically validated benchmarks [112]. And in response to the high drug resistance caused by antigenic escape, Mergen et al. established a framework for the *de novo* design of binders (DNBs) targeting tumor surface antigens, successfully engineering high‐affinity domains for EGFR and BCMA. These AI‐generated binders, whose length was set to 62AA, demonstrate functional performance—including cytotoxicity, cytokine secretion, and proliferative capacity—comparable to comparable to benchmarked scFvs currently used in clinical trials. A pivotal advancement of this platform is the utilization of de novo to precisely target the BCMA‐R27P mutation, which confers resistance to the bispecific antibody teclistamab, thereby showcasing the potential of the platform for rapid development (within an 8‐week timeframe) to overcome antigen escape [59].

While generative AI workflows provide a scalable framework for diversifying binder specificity, the de novo field concurrently advances through a structurally deterministic path: the optimization of established, hyperstable scaffolds that leverage high‐order thermodynamic stability for superior clinical performance. As early as 1999, the development of α3D—a complex 73‐residue three‐helix bundle—demonstrated that researchers could construct synthetic proteins with atomic‐level accuracy and exceptional thermodynamic stability entirely independent of natural templates, relying solely on physicochemical principles such as precise hydrophobic core packing, electrostatic programming, and capping motifs [113]. Derived from this de novo‐designed α‐helical bundle, D‐domains, a variant of α3D, referred to throughout as Dd‐a3D, possess the requisite properties for modular targeting agents: they are compact (roughly 1/3 the size of an scFv), single‐domain structures that lack disulfide bonds and N‐linked glycosylation. By incorporating these D‐domains into CAR‐T cells, researchers observed that, compared with traditional scFvs, these de novo‐designed domains—owing to their extreme folding stability and compact geometry—significantly reduced tonic signaling and delayed cell exhaustion [54]. Furthermore, through targeted engineering, this domain was specialized to engage BCMA, directing the potent elimination of BCMA‐expressing multiple myeloma cells [55]. This development, called anitocabtagene autoleucel (formerly CART‐ddBCMA, an autologous anti‐BCMA CAR T‐cell therapy with a novel synthetic D‐domain binder), has been robustly validated in the iMMagine‐1 (NCT05396885) clinical trial. Anito‐cel demonstrated exceptional efficacy in RRMM patients (97–100% ORR; ≥89% MRD negativity), notably without the delayed neurotoxicity—such as Parkinsonism—that limits other BCMA‐targeted CAR‐T therapies [56, 57]. Currently, the efficacy and safety of anito‐cel will be evaluated against the standard of care in the randomized, open‐label, Phase 3 iMMagine‐3 trial (NCT06413498). In summary, de novo protein design addresses the inherent limitations of natural antibody fragments in receptor engineering by providing recognition modules with superior biophysical stability.

#### 3.2.2 De Novo‐Engineered Protein Logic Gates for Programmable Immune Cell Regulation

Beyond structural modifications, Co‐LOCKR is a de novo‐designed protein switch that executes Boolean logic operations (AND, OR, NOT) on cell surfaces. Designed using highly orthogonal synthetic protein components, the system integrates multiple antigen inputs to program T‐cell activation. This approach allows T cells to precisely distinguish tumor cells from healthy tissues based on specific antigen combinations—such as requiring two markers (AND) or avoiding a safety marker (NOT)—thereby broadening the targeting scope while minimizing off‐target toxicities [60].

Designed from the perspective of logic gates, Chen et al. explored the application of de novo biological functional modules within the context of ACT, enabling the precise and programmable regulation of immune cell behavior. Utilizing Rosetta software and the HBNet algorithm, the researchers designed de novo heterodimers (DHDs) based on parametrically defined helical bundles. These de novo components leverage atom‐level precise internal hydrogen‐bond networks (HBNets) to ensure exceptional binding specificity and molecular orthogonality, effectively minimizing crosstalk with endogenous cellular proteins. Building upon this highly modular foundation, the team constructed a competitive protein logic gate system, CIPHR. In application, researchers engineered a de novo protein AND logic gate to precisely downregulate TIM3, a checkpoint receptor linked to T‐cell exhaustion. This system employs two modular monomers, sequence‐specific transcription activator‐like effector (TALE) DNA‐binding domains and the Krippel‐associated box (KRAB) repressor domain, that cooperatively assemble into a functional repressor complex via designed hydrogen‐bond networks only when both inputs are concurrently present, demonstrating the efficacy of posttranslational logic gates in mitigating cellular exhaustion and enhancing the persistence of engineered immune cells [61]. Taken together, this de novo designed orthogonal and programmable framework establishes a structural and functional foundation for the integration of synthetic logic gates into therapeutic design, facilitating the development of next‐generation ACT characterized by enhanced safety and sophisticated multi‐input control.

#### 3.2.3 De Novo‐Engineered Antigen Recognition Modules for Adoptive Cell Therapies

Given that the recognition of peptide‐MHC (pMHC) complexes by T‐cell receptors (TCRs) is a fundamental prerequisite for T‐cell activation and holds paramount importance for T‐cell‐based cancer immunotherapy, de novo design strategies have been increasingly employed to construct artificial proteins with high structural precision and functional specificity targeting these complexes [2, 114, 115, 116, 117].

First, regarding the methodological framework, Liu et al. [118] utilized RFdiffusion to design proteins capable of binding pMHC‐I by establishing extensive contacts with the target peptide. Based on experimentally determined or computationally predicted pMHC structures, they successfully generated specific binders for 11 distinct pMHC targets. Furthermore, integrating these designed proteins into chimeric antigen receptors (CARs) successfully induced peptide‐specific T‐cell activation for 8 of these targets. Second, focusing on integrated design pipelines, Johansen et al. developed small binding proteins (minibinders, miBds) targeting the tumor antigen NY‐ESO‐1 (SLLMWITQC)/HLA‐A*02:01. Their approach utilized a robust computational workflow encompassing scaffold generation via RFdiffusion, sequence optimization with ProteinMPNN, and interface prediction and ranking using AlphaFold2 [58]. Third, concerning conformational biomimicry, HouseHolder et al. [119] constructed modular TCR‐mimetic proteins (TCRms), with an emphasis on the precise replication of the native TCR's docking orientation rather than merely bypassing the MHC to recognize the peptide. Crystal structure analysis further validated their exquisite peptide‐recognition capabilities.

In terms of logical progression, the work by Liu et al. [118] systematically demonstrates an AI‐driven protein design framework that is scalable across diverse pMHC‐I contexts. Johansen et al. advanced this by translating binders into functional CAR modules for potent tumor cell killing. Finally, Householder et al. [119] focused on the spatial mechanics of recognition, ensuring that artificial proteins emulate the specific diagonal binding mode of natural TCRs to achieve highly controllable immune recognition.

While the application of de novo design in adoptive cell therapy has predominantly focused on CAR‐T cells, recent research has expanded this paradigm to CAR‐NK cells. Investigators engineered mesothelin‐specific CAR‐NK cells to secrete the de novo‐designed protein Neo‐2/15, which is generated by the Baker Lab [48], leveraging it to activate NRF1‐mediated mitochondrial metabolic enhancement. This strategy effectively counteracts immunosuppression within the solid TME, thereby significantly bolstering the persistence and cytotoxic efficacy of CAR‐NK cells against pancreatic and ovarian cancers [120].

### 3.3 Modulating Signal Pathways

Intercellular signal transduction can be conceptualized as a sophisticated biological communication protocol. Within the framework of cell therapy, conventional therapeutic interventions primarily rely on the administration of exogenous cytokines, co‐stimulatory agonists, and immune checkpoint modulators, or the modification of receptors derived from natural templates [121, 122, 123, 124, 125]. However, these traditional approaches are often hampered by a lack of spatiotemporal specificity, making it difficult to precisely regulate cell‐based drugs within complex microenvironments.

To overcome these limitations, de novo protein design enables the programmed regulation of how cells perceive and integrate extracellular signals. By engineering high‐affinity interfaces directed at the three‐dimensional structures of targets, utilizing conformational switches to achieve conditional activation, and ensuring high selectivity for specific receptor subtypes, this approach facilitates the development of intelligent cellular therapeutics. These advancements provide a robust foundation for next‐generation therapies characterized by precise conditional triggering and sophisticated signal processing capabilities.

#### 3.3.1 Spatial and Geometric Control

To leverage de novo protein design for the precise modulation of downstream cellular signaling, it is essential to programmatically manipulate the valency and spatial arrangement of receptors using modular, custom‐designed scaffolds.

A de novo protein design strategy has been developed to enable the programmable modulation of cellular signaling pathways by precisely engineering receptor organization. This approach is founded on the generation of hyperstable, high‐affinity miniprotein binders designed exclusively from the three‐dimensional structures of target receptors. By fusing these binders to modular, cyclic homo‐oligomeric scaffolds, the platform allows for the quantitative arrangement of receptor valency (up to eight subunits) and spatial spacing, thereby directly regulating downstream signal intensity. The effectiveness of this design strategy has been rigorously validated across 12 structurally diverse protein targets [126]. For instance, this platform was successfully applied to the regulation of fibroblast growth factor receptor (FGFR) signaling. By controlling the spatiotemporal clustering of the receptors, the researchers demonstrated that FGFR activation—characterized by MAPK phosphorylation and Ca^2+^ mobilization—is critically dependent on the specific geometric configuration and valency of the receptor complex [62, 127]. This modular architecture thus provides a robust and universal framework for rewiring biological communication protocols.

Notch signaling is a highly conserved, contact‐dependent mechanism that regulates cell fate and development through direct transmembrane ligand‐receptor interactions [128]. While iPSC‐derived T cells are a major trend in “off‐the‐shelf” adoptive cell therapy, their large‐scale production is hindered by the requirement of mechanical force (usually provided by cell‐associated ligands) for Notch activation [129]. To overcome this, researchers developed de novo soluble agonists designed to promote cell–cell bridging and cluster Notch receptors at cell synapses. By engineering specific spatial organizations, these scaffolds trigger receptor phosphorylation and signaling without the need for physical pulling forces. The researchers utilized de novo‐designed helical bundles with initially cis‐oriented termini, which were subsequently reconfigured into trans‐presenting complexes by radially extending the scaffold arms with rigid helical repeat proteins [130]. Ultimately, it is demonstrated that precise spatial clustering alone is sufficient to trigger Notch proteolysis, effectively resolving the fundamental scientific challenge of decoupling receptor activation from its natural requirement for mechanical pulling force.

#### 3.3.2 Dynamic Switches and Biochemical Logic

Dynamic switching and conditional triggering strategies aim to endow signaling pathways with logical response capabilities in specific spatiotemporal dimensions, such as the tumor microenvironment, by converting biochemical stimuli (such as phosphorylation, pH, or protein cleavage) into conformational switching or shielding removal of proteins.

In terms of the mechanism of remodeling signaling pathways, a notable advancement is the development of phosphorylation‐induced protein switches (PIPS), which utilize RFDiffusion and multistate ProteinMPNN to encode a kinase‐responsive conformational toggle. By embedding a phosphorylation‐sensitive motif (e.g., the PKA‐recognized RRAS sequence) within a buried helical core, researchers successfully engineered a switch that transitions from a closed autoinhibited state to an open active state upon phosphorylation. This design effectively emulates natural signaling cascades by coupling a biochemical input to a structural output [63].

In the T‐cell activation cascade, the phosphorylation of the immunoreceptor tyrosine‐based activation motif (ITAM) is the key initiation step for ZAP‐70 kinase recruitment [131]. Orchestrating the interaction between ITAM and its co‐regulators thus represents a fundamental strategy for regulating the intensity of the downstream immune response. Therefore, a study describes the de novo design of two classes of protein switches that dynamically couple kinase phosphorylation with protein‐protein association. Specifically, these switches consist of de novo‐designed four‐helix bundles that cage functional motifs within their hydrophobic cores, utilizing steric hindrance or electrostatic repulsion—triggered by co‐regulator binding or kinase phosphorylation—to disrupt internal energetic competition and drive conformational activation. In the first architecture, protein association serves as a prerequisite for kinase‐mediated phosphorylation, whereas in the second, kinase activity directly drives the association process. These highly reversible switches achieve up to a 40‐fold activation in cellular environments, successfully transducing the kinase activity of the ITAM (immunoreceptor tyrosine‐based activation motif), a central hub in T‐cell signaling [132].

Another study proposes a de novo peptide masking workflow to enable the rapid conditional activation of miniprotein binders by extending their C‐terminus with a masking helix that reversibly blocks the binding interface through steric hindrance. Using the EGFR antagonist EGFRn_mb as a lead model, the candidate M3 achieved an over 1,000‐fold reduction in affinity, allowing for conditional activation via tumor‐specific proteases (e.g., matrix metalloproteinase 2/9) or external light (365 nm) to precisely inhibit the MAPK/ERK cascade while mitigating systemic toxicities across multiple targets, including EGFR, FGFR2, and IL7Rα [64]. Furthermore, the development of pH‐sensitive binders provides an alternative layer of control by exploiting the ubiquitous acidity of the TME. By computationally encoding histidine‐based repulsion or core‐destabilization, these de novo proteins respond to specific pH changes. Notably, the integration of pH‐sensitive designs into lysosomal trafficking systems moves beyond simple signaling blockade toward catalytic protein degradation. This strategy can significantly enhance the potency of immunotherapies against targets such as TNFR2, EphA2, and PCSK9 by actively depleting the pathogenic proteins [133].

#### 3.3.3 Precision Recognition and Subtype Selectivity

Precise identification and subtype selectivity focus on using atomic‐level precision interface design to overcome the inherent signal confusions of natural ligands, achieving highly specific and exclusive identification among receptor subtypes with highly similar sequences.

Frizzled (Fzd) receptors activate the Wnt/β‐catenin signaling pathway to drive T‐cell exclusion, effectively transforming tumors into immunologically cold phenotypes resistant to immune checkpoint inhibitors, thereby serving as a pivotal target for overcoming immunotherapy resistance [134, 135]. To address the inherent promiscuity of the Wnt signaling pathway, researchers constructed a modular, Fzd7‐specific Wnt surrogate by fusing de novo‐designed receptor‐binding domains, resulting in a potent affinity of Kd < 2.3 nM for the Fzd7 subtype [29].

De novo design strategies are similarly applied to regulate the differential binding of integrins, which are αβ heterodimeric receptors that act as key mediators for immune cell adhesion and signaling. By mediating ECM or intercellular interactions, specific integrins—such as LFA‐1(αLβ2) and VLA‐4(α4β1)—orchestrate innate and adaptive responses ranging from immune synapse formation and T‐cell activation to memory development [136, 137, 138, 139, 140]. In pathological contexts, the RGD‐binding integrins αvβ6 and αvβ8 have emerged as clinically validated targets for cancer and fibrosis. As integrins αvβ6 and αvβ8 bind their natural L‐TGF‐β1 and β3 ligands with low nM affinity, researchers leveraged the crystal structure of the αvβ6–L‐TGF‐β3 peptide complex and employed the Rosetta software to design de novo proteins capable of binding integrins. As a result, the inhibitors B6_BP_dslf and B8_BP_dslf achieved picomolar affinity while maintaining high selectivity for αvβ 6 and αvβ8, respectively, demonstrating precise discrimination between these homologous integrin subunits [65].

### 3.4 Engineering Checkpoint Inhibition

Immune checkpoints are a repertoire of regulatory molecules on immune cells that modulate the intensity of immune responses [141, 142]. However, tumor cells often exploit these pathways by overexpressing specific inhibitory ligands, which interact with checkpoint receptors to suppress immune activity and facilitate immune evasion [143, 144, 145]. Immune checkpoint blockade (ICB) therapies utilizing PD‐1/PD‐L1 and CTLA‐4 antibodies face limitations like high costs and poor TME penetration (∼150 kDa), resulting in uneven efficacy [146, 147, 148, 149]. Additionally, life‐threatening immune‐related adverse events (irAEs), such as hepatitis and endocrine dysfunction, significantly compromise patient safety and clinical outcomes [150, 151, 152, 153]. Advancements in de novo protein design research are establishing a new paradigm through their successful application to immune checkpoint inhibitors (ICIs). Recognizing that the binding interfaces of critical receptors like CTLA‐4 and PD‐L1 often feature convex topographies—which are difficult to target with high affinity using standard Rosetta‐based scaffolds—researchers engineered specialized five‐helix bundle helical concave scaffolds for precise geometric complementarity. To achieve this, helical and loop fragments were modularly assembled into helix‐turn‐helix units to form the five‐helix bundles, followed by backbone diversification using the Rosetta HybridizeMover and curvature filtering via random sample consensus (RANSAC) to ensure optimal fit for the receptors’ convex surfaces [66].

To precisely quantify PD‐L1 expression, PD‐L1‐3 was engineered as an ultrahigh‐affinity miniprotein with sub‐picomolar (< 1 pM) binding and exceptional hyper‐stability. Unlike antibody tracers requiring days for systemic clearance, PD‐L1‐3 exhibits notable rapid biphasic clearance kinetics (with fast‐ and slow‐phase half‐lives of 1.016 and 15.09 min, respectively), leading to a swift reduction in blood background signals. Combined with its ultra‐high affinity, these characteristics allow tumor tissues to reach detection thresholds as early as 5 min postinjection and achieve peak accumulation within 30 min. Furthermore, it combines deep tissue penetration with prolonged tumor retention (>4 h), surpassing cyclic peptides that typically show reduced accumulation after 90 min. Explicitly engineered for noninvasive diagnostics, PD‐L1‐3 demonstrated in first‐in‐human studies that its maximum standardized uptake value (SUVmax) correlates strongly with clinical PD‐L1 levels, accurately distinguishing high‐expression (SUVmax = 8.8) from low‐expression (SUVmax = 1.4) tumor profiles to monitor its spatial and temporal dynamics [67].

Beyond conventional inhibition, *de novo* principles have advanced toward active protein clearance, a process that actively reduces target protein density via lysosomal pathways, through multivalent targeting chimeras (multi‐TACs). Unlike traditional degradation strategies that rely on specific lysosome‐targeting receptors, multi‐TACs (such as the optimized GG56) utilize a copolymerized architecture of PD‐L1 inhibitors and acid‐responsive monomers to trigger tumor‐specific degradation. By precisely tuning valency and spacer length, these chimeras facilitate adsorption‐mediated endocytosis and lysosomal degradation of PD‐L1. This platform not only reduces checkpoint density on tumor surfaces but also acts as a versatile vehicle for combinatory chemoimmunotherapy, demonstrating significant tumor suppression in aggressive melanoma and breast cancer models [68].

## 4 Challenges and Prospects

While de novo‐designed proteins offer immense therapeutic potential, clinical translation requires overcoming safety, delivery, and cost barriers. As synthetic, nonnatural molecules, ensuring low immunogenicity and high targeting precision remains a paramount prerequisite for their successful medical application.

### 4.1 Immunogenicity

Xenogeneic immunogenicity refers to the ability of exogenous proteins or peptides to elicit an immune response in the host. Research indicates that the primary triggers for such immune responses in proteins include structural xenogeneity, specific amino acid sequences that may create novel T‐cell epitopes, and unique glycosylation patterns (e.g., nonhuman glycan structures if produced in nonhuman expression systems) [154, 155, 156, 157].

#### 4.1.1 Immunogenic Superiority of De Novo Designed Proteins

Based on various studies regarding de novo‐designed proteins, the immunogenicity profiles of these molecules demonstrate a potential that is significantly superior to natural protein variants. This advantage is primarily attributed to their idealized physicochemical properties and controllable sequence space [87].

First, from a physicochemical perspective, de novo proteins typically exhibit high thermodynamic stability and compact molecular dimensions. This stability is hypothesized to effectively inhibit protein degradation or aggregation, thereby reducing the pro‐inflammatory signals that trigger immune activation. Concurrently, predictive algorithms such as NetMHC indicate that, due to their significantly shorter sequence lengths, de novo binders harbor a lower absolute number of potential immunogenic epitopes across diverse HLA alleles compared with traditional scFvs [112]. Second, regarding sequence characteristics, the minimal homology between de novo proteins and endogenous counterparts (e.g., natural IL‐2) suggests that any elicited antibody response is unlikely to cross‐react with native cytokines, thus circumventing the risk of autoimmunity associated with the unintended neutralization of endogenous molecules [48].

Furthermore, the unique spatial conformations of these proteins may contribute to the robustness of their clinical efficacy. Observations from studies on mimetics such as 21h10 reveal that therapeutic potency remained largely unattenuated even in the presence of antidrug antibodies (ADAs). This phenomenon is hypothesized to occur either because immunodominant epitopes are located away from the receptor‐binding interface or because the high binding affinity of the de novo protein for its receptor favors target engagement despite the presence of bound antibodies [50]. Although the favorable positioning of these epitopes in current cases may have occurred somewhat fortuitously, it demonstrates the feasibility of using computational simulations to rationally redirect immune responses away from functional domains, thereby co‐optimizing immunogenicity and activity. Finally, clinical data from agents such as anito‐cel—characterized by rare ADA occurrences and an absence of delayed neurotoxicity—provide practical validation for the advantages of de novo architectures in minimizing sequence‐based liabilities and enhancing overall systemic safety [56, 57].

Despite these structural advantages, the immunogenicity of de novo proteins remains a latent concern inherent to any nonhuman‐derived system [87, 158]. Given the requirement for repeated systemic administration, the potential for complex immune responses across diverse patient populations necessitates rigorous clinical monitoring and the continued refinement of negative design strategies to ensure long‐term safety [87].

#### 4.1.2 Strategies for Minimizing Immunogenicity in De Novo Protein Design

To reduce the immunogenicity of de novo designed proteins, several strategies can be employed. Computational strategies for protein de‐immunization primarily rely on the precise identification and elimination of T‐cell epitopes. As the mainstream deep‐learning tool for MHC‐II, NetMHCIIpan‐4.0 achieves high‐precision predictions by integrating large‐scale peptide‐HLA binding data [159]. However, while it marks a significant improvement with a median FRANK (fractional rank) of 0.0351, it remains inherently less precise than MHC‐I methods like NetMHCpan‐4.1, which reaches a median FRANK of 0.0022 [159]. MARIA is a multimodal recurrent neural network that significantly improves the prediction of HLA class II antigen presentation by integrating diverse data, outperforming existing tools in identifying immunogenic epitopes for vaccines and cancer therapies (area under the curve = 0.89–0.92) [160]. These algorithms, combined with the Consensus methods provided by the Immune Epitope Database (IEDB), form the technical cornerstone of the rational control of immunogenicity in modern de novo protein design [161].

Beyond simple recognition, current protein de‐immunization methodologies now directly integrate immunogenicity scoring into the generative trajectory to facilitate precise modifications without compromising structural integrity. Besides, researchers frequently employ structural optimization frameworks such as EpiSweep. This algorithm utilizes integer linear programming (ILP) to identify optimal mutational schemes that maximize epitope deletion with minimal mutational cost, while strictly satisfying constraints for thermodynamic stability and functional folding [162]. DPO‐ProteinMPNN utilizes direct preference optimization (DPO) to retrain generative models, enabling the autonomous selection of sequences with lower predicted immune risk during the decoding phase [163]. At the same time, CAPE‐Beam is a novel decoding strategy for ProteinMPNN that minimizes cytotoxic T‐lymphocyte (CTL) immunogenicity by restricting sequence synthesis to human‐like kmers that are either predicted to avoid CTL presentation or are subject to central tolerance, while effectively preserving the protein's structural integrity [164].

Given the discrepancy between computational predictions and actual immune responses, would integrating experimental validation data—including in vitro T cell activation assays and immunogenicity assessments in animal models—enhance the predictive accuracy of these models? Currently, there is a lack of systematic research specifically focused on utilizing experimental feedback in a closed‐loop manner to improve algorithmic precision. Prevailing literature typically follows a unidirectional validation pattern: a novel immunogenicity prediction algorithm is proposed, and subsequently, experimental data are used to demonstrate that designs guided by this algorithm significantly reduce protein immunogenicity. For instance, in studies of the recombinant immunotoxin PE38, researchers utilized the IEDB algorithm to screen mutations and combined this with IL‐2 ELISpot assays for high‐resolution epitope mapping; this synergistic computational and experimental strategy ultimately achieved a 93% reduction in T cell epitope responses [165]. Similarly, in the development of recombinant Factor VIIa analogs, epitopes were identified via rational immunogenicity determination (RID) and validated through in vitro T‐cell activation assays, successfully reducing immunogenicity without compromising biological activity [166]. These validated examples demonstrate that while experimental validation is currently used primarily as a terminal indicator of algorithmic efficacy, this collaborative computational screening‐experimental validation paradigm has become a recognized and effective pathway for mitigating the immunogenic risks of both de novo and engineered proteins. However, we could envision a prediction–validation–feedback–optimization closed‐loop paradigm, where experimental outputs are systematically fed back to refine algorithmic parameters, thereby iteratively enhancing the predictive accuracy of immunogenicity for de novo‐designed proteins.

### 4.2 Off‐Target Toxicity

Off‐target toxicity, defined as unintended interactions between a synthetic protein and nontarget molecules, remains a primary concern in protein therapeutics as it may diminish efficacy or trigger systemic toxicity. In de novo protein design, mitigating these risks requires a dual‐pronged approach: negative design to prevent unintended conformations and specificity engineering to ensure precise molecular recognition. While there are currently no documented clinical cases of de novo‐designed proteins being used in tumor immunotherapy to identify and preemptively exclude all potential off‐target receptors or molecules, the fundamental principles validated in recent research provide a robust framework for such specificity.

At the computational design level, structural specificity is achieved by engineering “funnel‐shaped” energy landscapes. As demonstrated by Koga et al. [17], enforcing strict physical rules between secondary and tertiary motifs ensures that the target fold remains thermodynamically isolated from misfolded, potentially toxic decoy states. To further refine binding selectivity, researchers employ preorganized, shape‐complementary interfaces and explicit hydrogen‐bonding networks. A representative study by Tinberg et al. [167] showcased that de novo proteins can distinguish targets from structurally nearly identical molecules (e.g., digoxigenin vs. progesterone) with picomolar affinity, providing a robust framework for high‐precision immunotherapy. The proficiency of de novo protein design in engineering high‐affinity, selective binders is further evidenced by previously discussed immunotherapeutic candidates, including Neo2/15, des03, B6_BP_dslf, and B8_BP_dslf. To enhance systemic safety, these high‐precision interfaces can be further integrated with conditional activation strategies—such as the protease‐cleavable peptide masks previously detailed in Section 3.3.2—which sterically seal the active site until it is triggered within the tumor microenvironment [138]. Finally, the safety profile must be rigorously validated through a multitier pipeline. High‐throughput screening (HTS) in vitro assays are employed to evaluate cross‐reactivity across diverse cell lines and pathways. Subsequently, preclinical animal models (e.g., rodents and nonhuman primates) are essential to assess systemic off‐target interactions and long‐term organ toxicity that cannot be captured by computational or cellular models.

### 4.3 Synthetic Protein Delivery

The delivery of therapeutic proteins into the TME remains a multifaceted challenge. While traditional biologics often require postdiscovery modifications to withstand the TME, de novo protein design offers a paradigm shift: integrating delivery solutions directly into the protein's primary structure during the initial design phase.

#### 4.3.1 Protease Stability and Structural Immunity

The in vivo performance of computationally designed proteins depends not only on their intrinsic stability but is also profoundly influenced by complex protein–protein interaction (PPI) networks. Within such networks, endogenous proteases act as critical degradation nodes, intercepting therapeutic proteins by recognizing their exposed flexible regions. Rather than employing external stabilizers, the de novo strategy prioritizes structural immunity at the design stage. By computationally optimizing dense hydrophobic core packing and minimizing flexible regions, researchers have engineered hyper‐stable miniprotein scaffolds that exhibit extreme thermal (its CD spectrum is essentially unchanged at 95°C) and proteolytic resistance (trypsin and chymotrypsin) [168]. This stability ensures that the therapeutic payload remains intact within the harsh TME without the need for complex exogenous protection.

However, beyond resisting proteolytic interception, de novo proteins still encounter challenges from other complex nonspecific interactions within the dynamic PPI network, such as protein corona formation or physical sequestration [169]. While structural immunity addresses the enzymatic degradation aspect of these network‐driven hurdles, the systemic difficulties associated with deep tissue penetration and the heterogeneity of the immune microenvironment pose additional barriers, which will be discussed in detail in Section 4.2.3.

#### 4.3.2 Half‐Life and Systemic Circulation

While the small size of de novo proteins facilitates tissue penetration, it often leads to rapid renal clearance. For instance, the pSTAT5 signaling response to Neo‐2/15 (a de novo IL‐2 mimic) was markedly reduced after 3 h and undetectable after 8 h in mice, owing to its small molecular weight [48]. This PK limitation is being resolved through de novo engineering. To extend systemic circulation, chemical modifications such as PEGylation are universally applied [170, 171]. Silva et al. [48] reckoned that half‐life can be extended by incorporating site‐specific PEGylation (via engineered cysteines) or fusing the scaffold to Fc regions, DARPins, or other de novo‐designed binding proteins during the sequence definition phase, maintaining pristine binding kinetics while prolonging blood residence time [172]. Moving forward, integrating clearance considerations as a parameter during the ab initio design phase will be essential to ensure that therapeutic longevity is an intrinsic property of the protein scaffold rather than an extrinsic addition.

Complementing these molecular‐level modifications, the encapsulation of de novo proteins within exosomal vehicles offers a multiscale delivery strategy. At the systemic level, exosomes leverage surface‐expressed CD47 signals (e.g., iExosomes) to antagonize phagocytosis by monocytes and macrophages [173]. To preserve the structural integrity of designed proteins, technologies like EXPLOR utilize optically reversible protein interactions to achieve active, nondestructive loading during exosome biogenesis, ensuring that engineered binding kinetics remain pristine [174]. Furthermore, by utilizing pathways such as the L‐domain for packaging, exosomes have demonstrated a superior capacity for traversing substantial biological barriers, including the blood–brain barrier, to achieve functional intracellular delivery [175].

#### 4.3.3 TME Barrier

The tumor microenvironment itself imposes additional delivery barriers. The dense ECM, irregular vasculature, and heterogeneous redox conditions hinder deep tissue penetration [176]. This is particularly problematic in solid tumors and is further exacerbated in diseases like glioblastoma, where the blood–brain barrier poses an additional layer of protection against therapeutic entry [177, 178]. Therefore, by computationally designing geometrically matched beta strands to complement the exposed beta‐sheet edges of the human transferrin receptor (hTfR), researchers developed hyperstable, nanomolar‐affinity binders that successfully cross the blood–brain barrier for targeted brain drug delivery [179].

Regarding the penetration problem in immunotherapy for solid tumors, a study demonstrates the successful de novo construction of β‐barrel membrane protein channels with precisely programmed pore diameters of 5–10 Å, validating their high‐efficiency water permeability and rigorous size‐exclusion characteristics. These findings offer a novel biophysical strategy to address the penetration barrier in solid tumor immunotherapy [180]. High interstitial fluid pressure (IFP) within the tumor microenvironment, driven by vascular leakage and impaired lymphatic drainage, represents a major physical impediment to the deep infiltration of T‐cells and monoclonal antibodies. Looking forward, the exceptional water transport capacity of these de novo pores could be leveraged as a synthetic biology toolkit to modulate local osmotic and hydrostatic gradients. By engineering these channels for expression within tumor stromal cells or via targeted delivery vectors, it may be possible to facilitate fluid efflux and actively dissipate IFP. Such physical remodeling of the tumor landscape would enhance the convective transport of therapeutic agents and the active migration of immune cells, ultimately improving the clinical efficacy of cell‐based therapies in recalcitrant solid malignancies.

**FIGURE 4:**
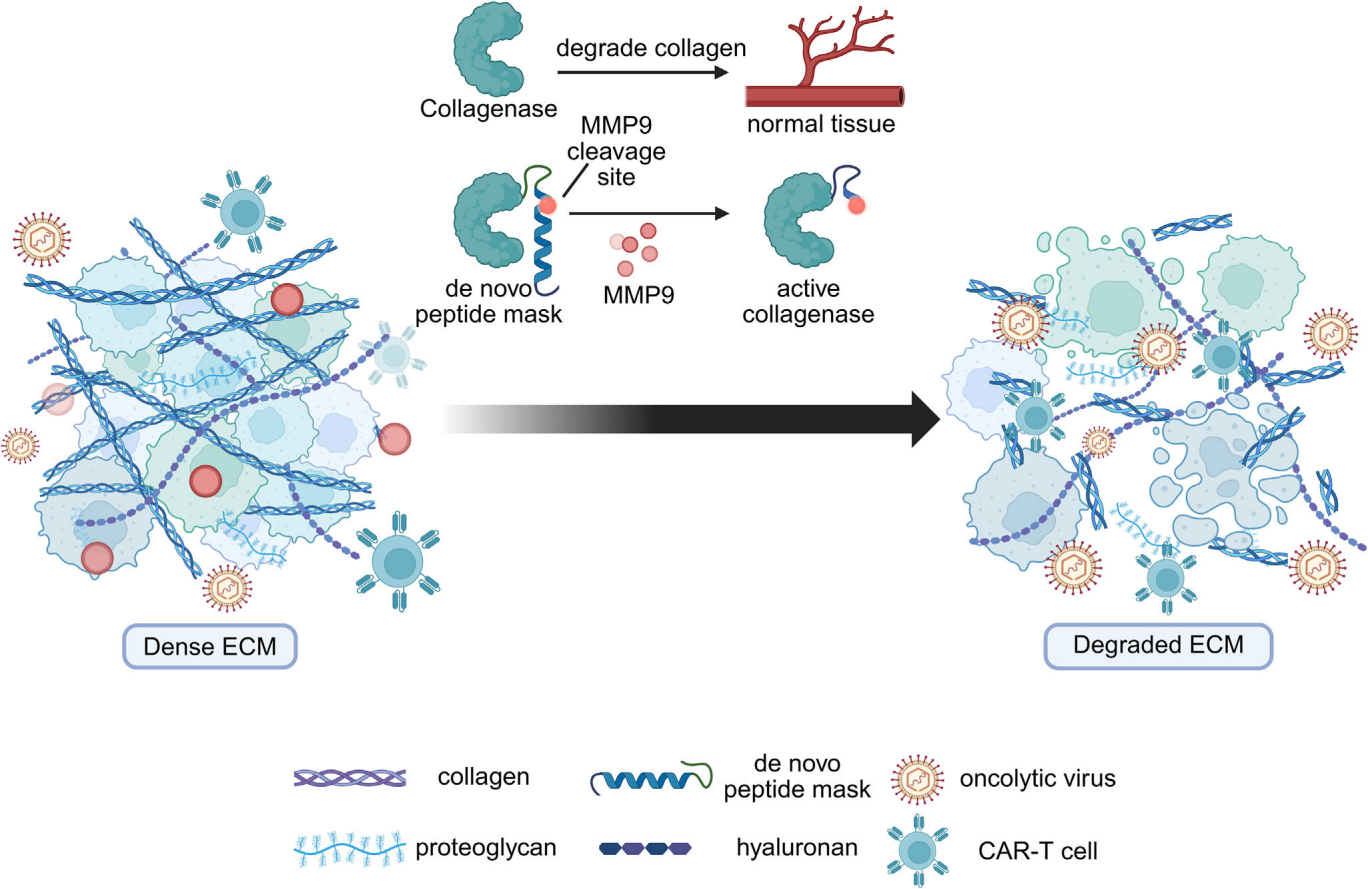
Schematic of the de novo designed collagenase masking strategy for targeted tumor extracellular matrix (ECM) degradation. While native collagenase often exhibits nonspecific toxicity by attacking collagen in normal tissues, this approach employs a de novo engineered peptide mask to sterically hinder the enzyme's peptidase site, maintaining it in an inactive state during systemic circulation. Upon reaching the TME—where Matrix Metalloproteinase‐9 (MMP9) is highly overexpressed, as frequently observed in pancreatic cancer—the mask is selectively cleaved and released, triggering localized collagen degradation. This targeted remodeling of the dense ECM architecture significantly enhances the intratumoral infiltration and therapeutic efficacy of co‐administered oncolytic viruses and CAR‐T cells (figure was created with Biorender.com).

The dense ECM of solid tumors forms a formidable physical barrier against protein therapeutics, yet the clinical utility of exogenous collagenases to degrade this matrix is severely constrained by off‐target toxicity and the inherent complexity of de novo catalytic design [181, 182, 183, 184, 185, 186, 187, 188]. To circumvent these obstacles, we propose a conditionally active platform utilizing a de novo masking peptide, which is discussed in Section 3.3.2 (Figure 4) [64]. By sterically blocking the enzyme's binding interface with a masking helix tethered via a tumor‐protease‐cleavable linker (e.g., MMP‐responsive), the collagenase remains sequestered in systemic circulation—achieving a potential 1000‐fold reduction in off‐target affinity. Upon reaching the protease‐rich tumor microenvironment, the mask is selectively released, enabling localized ECM remodeling and facilitating the deep infiltration of therapeutic agents into the tumor core.

### 4.4 Cost

In the field of protein engineering, the shift in technical paradigms—from utilizing natural templates to de novo synthesis—has led to a significant evolution in research and development (R&D) costs. Traditional protein engineering costs are primarily driven by the high amortized R&D expenditure per candidate, resulting from prolonged, resource‐intensive wet‐lab screening cycles and the technical risks inherent in balancing activity and stability within limited evolutionary templates.

In contrast, de novo protein design explores nonnatural sequence spaces via algorithms, exhibiting a cost structure characterized by high computational intensity compared with traditional engineering. During the backbone generation stage, diffusion models such as RFdiffusion have significantly reduced construction times; for instance, generating a 100‐residue protein takes approximately 11 s on an NVIDIA A4000 GPU, nearly 50 times faster than the early RF Hallucination method (∼8.5 min) [30]. Subsequently, for sequence design, ProteinMPNN NIM has emerged as the most cost‐effective component of the pipeline due to its lack of complex customization requirements. With a modest VRAM requirement of just 3 GB, the system is optimized to operate on a single NVIDIA GPU possessing a compute capability of 7.0 or higher.

However, structural validation remains the most significant computational burden in the current pipeline. Validation models, exemplified by AlphaFold 2, require approximately one week of training on 128 TPU v3 cores. In practice, predicting a long‐chain protein such as a collagenase precursor (2500 residues) can take up to 18 h on a single V100 GPU. Moreover, these tasks frequently outstrip the memory limits of individual cards, necessitating the use of multi‐GPU clusters (e.g., 4 GPUs) for collaborative inference due to the O(n^2^) complexity of the underlying algorithms [36]. To overcome the barriers of multistep design, cutting‐edge multimodal models like ESM3 (98 billion parameters) aim to achieve unified programming of sequence, structure, and function at the atomic level. Yet, this versatility rests on a massive computational foundation; ESM3 consumed approximately 1.07 × 10^24^ FLOPs during pretraining on 2.78 billion proteins and 771 billion unique tokens [189]. This reflects the extreme computational challenges inherent in the evolution of de novo design toward large‐scale foundational models.

The cost‐related challenges of de novo protein engineering can be categorized into three primary stages. First, the synthesis and expression of novel sequences often require expensive solid‐phase peptide synthesis (SPPS) or complex recombinant systems, where optimizing translation and posttranslational modifications remains a resource‐intensive bottleneck [13]. Second, the necessity for high‐purity isolation, utilizing costly techniques like affinity chromatography and high‐performance liquid chromatography (HPLC), presents significant hurdles for both laboratory purification and industrial scalability [190]. Third, the inherent lack of physiological stability in many designed proteins demands additional, expensive formulation strategies, such as PEGylation or nanoparticle encapsulation, to ensure adequate bioavailability [191].

Finally, the economic burden of wet‐lab validation is being mitigated through tiered AI‐driven screening and high‐throughput synthesis. Platforms like Twist Bioscience have leveraged silicon‐based chip technology to reduce the starting price of gene fragments to $0.09/bp. By utilizing systematic optimization with up to 70 Golden Gate sites, the OMEGA (Oligo‐based multiplexed efficient gene assembly) platform enables the parallel assembly of de novo genes up to 2.6 kb from low‐cost oligopools, slashing per‐gene costs to $1.50–$14 compared with over $50 required by traditional fragment synthesis for long proteins like Cas9 [192]. By integrating self‐consistency checks (e.g., filtering for pLDDT [predicted Local distance difference test] >70, which means the main structure is reliable, and low predicted aligned error) before synthesis, researchers can prune 90% or more of nonviable designs in silico, ensuring that expensive experimental resources are concentrated solely on the most promising candidates.

### 4.5 A Vision for De Novo Designed Multiepitope Cancer Vaccine

Cancer vaccines represent a rapidly evolving field in tumor immunotherapy. To effectively address tumor heterogeneity and mitigate the risk of antigenic escape—a process where clonal variants evade surveillance by downregulating single targets—the field has shifted toward multiepitope vaccines designed to broaden immunological coverage [193, 194]. However, current clinical candidates, including BNT111, VGX‐3100, and Provenge, predominantly utilize these epitopes in their native form as linear long peptides [195, 196, 197]. A critical limitation of this paradigm is the treatment of antigens as simple linear sequences; this approach frequently leads to suboptimal structural stability and unpredictable intracellular processing [198]. Such structural deficiencies often result in an imbalanced immune response, characterized by a marked predominance of CD4^+^ T‐cell activation over the critical CD8^+^ effector response [194].

**FIGURE 5:**
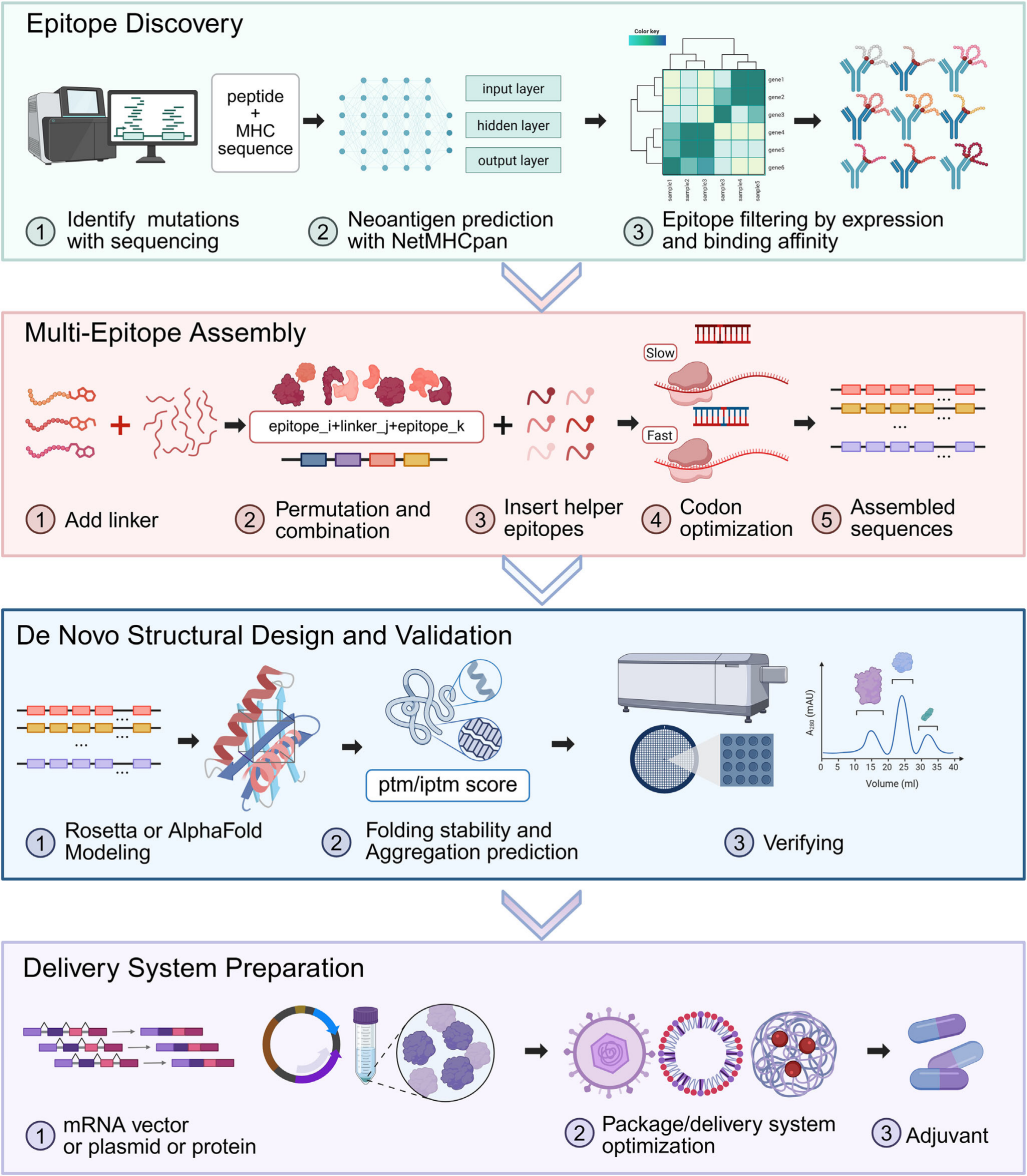
Workflow of de novo designed multiepitope vaccine development. This closed‐loop pipeline illustrates the transformation of biophysical constraints into potent therapeutic candidates through four integrated stages: Epitope Discovery begins with identifying mutations via sequencing, followed by neoantigen prediction using NetMHCpan to analyze peptide‐MHC binding through deep learning layers, and final filtering based on expression levels and binding affinity. Multiepitope assembly involves the addition of linkers and the permutation of epitopes to form complex linear constructs, which are further enhanced by helper epitopes, codon optimization, and physicochemical profiling. De novo structural design and validation employ Rosetta or AlphaFold for 3D modeling, utilize ptm/iptm scores to predict folding stability and aggregation tendencies, and conclude with experimental verification via methods such as size‐exclusion chromatography. Finally, delivery system preparation involves selecting appropriate vectors—such as mRNA (equipped with 5’Cap and PolyA tail), plasmids, or proteins—and optimizing the delivery package with specific adjuvants to ensure robust immune activation (figure was created with Biorender.com).

To transcend these clinical hurdles, we propose a transformative synthesis paradigm for multiepitope cancer vaccines centered on de novo protein engineering. This approach fundamentally diverges from traditional methodologies by identifying immunogenic “hot‐spot” motifs within natural or predicted antigens and subsequently re‐architecting them into computationally designed de novo protein backbones. By transitioning from the direct use of native sequences to these synthetic scaffolds, we aim to address the inherent structural instability and delivery constraints that have historically compromised the efficacy of tumor antigens. Within this conceptual framework, we envision that de novo engineering can systematically overcome the limitations of conventional vaccines through four synergistic strategies: (1) precision surface presentation, where de novo strategies like Scaffold Grafting plant epitopes into hyper‐stable, nonnatural scaffolds (e.g., FN3 domains) to ensure rigid exposure and maximize receptor accessibility [199, 200, 201, 202]; (2) superior immunogenicity control, achieved by engineering binders with minimal human homology and utilizing computational simulations to redirect immune responses away from functional domains, thereby mitigating autoimmunity [203]; (3) modular structural integration, which employs tandem fusion and modular assembly with computationally optimized linkers (e.g., GGGGSn) to coordinate multiple heterologous epitopes while preventing junctional neo‐epitopes [204, 205]; and (4) AI‐powered backbone generation, leveraging frontier generative models like RFdiffusion and ProteinMPNN to create entirely novel architectures tailored for specific tumor epitopes. As a synthesis of these advancements, the workflow illustrates our envisioned integrated de novo engineering workflow, a closed‐loop pipeline that transforms complex biophysical constraints into intrinsic properties of next‐generation, high‐potency cancer vaccines (Figure 5).

### 4.6 Clinical Test

Despite the vast theoretical potential of de novo protein design for cancer immunotherapy, definitive clinical breakthroughs remain elusive. Encouragement is drawn, however, from nononcology successes, such as ultrastable viral vaccine scaffolds, which demonstrate that computational simulations can indeed survive complex physiological environments. The arduous transition from in silico innovation to bedside application is exemplified by NL‐201, a computationally designed IL‐2/IL‐15 receptor agonist engineered to selectively activate CD8+ T and NK cells via the IL‐2Rβ/γ complex. By intentionally avoiding the IL‐2Rα subunit, NL‐201 aimed to eliminate the severe toxicities of traditional IL‐2 therapies. Yet, the clinical reality proved humbling: the Phase 1 trial (NCT04659629) concluded without publicly disclosed efficacy data, underscoring the persistent translational gap for purely synthetic functional proteins [49].

In response to these setbacks, the focus is pivoting toward therapeutic binders that prioritize early‐phase safety and optimal biodistribution. A current representative case is the PD‐L1‐3 miniprotein, which was mentioned before, an ultrahigh‐affinity binder designed to target the PD‐1/PD‐L1 interface. This candidate is currently entering the initial phase of clinical investigation, with recruitment ongoing for a first‐in‐human (FIH) study [67]. Crucially, while fully synthetic functional proteins encounter significant hurdles, de novo‐designed protein scaffolds have already reached late‐stage clinical milestones.

A preeminent example is GBP510, which leverages the de novo I53‐50 nanoparticle scaffold, a 28 nm icosahedral complex self‐assembled from 120 synthetic subunits, to multivalently display 60 copies of the SARS‐CoV‐2 RBD [205, 206]. By mimicking the repetitive structural motifs of virus‐like particles (VLPs), this platform significantly amplifies immune activation. Clinical studies have confirmed that the AS03‐adjuvanted regimen induces exceptionally high antibody titers (IgG up to 2599.2 BAU/mL); while local reactogenicity (such as pain) is slightly higher, it remains safe and manageable [207]. The Phase III trial (NCT05007951) further established its superiority, with a neutralizing antibody GMT 2.93 times that of the ChAdOx1‐S group and protective efficacy sustained for over 6 months. This marks the successful pivotal clinical validation of computationally designed protein scaffolds [208].

To overcome the remaining hurdles, recent algorithmic advancements are introducing more robust validation patterns. Improved immunogenicity prediction is being achieved through deep learning models that integrate T‐cell epitope and HLA‐binding predictions, as discussed in 4.1 Immunogenicity, leveraging large‐scale human immune datasets and refined humanization strategies. Multiscale physiological simulations, combining molecular dynamics with systems biology, now incorporate tissue microenvironment factors and patient‐specific data to enhance de novo therapeutic distribution and activity predictions. Current clinical translation of de novo proteins is shifting toward a gradual validation pattern to mitigate the risks observed in early pure‐synthetic trials. Specifically, hybrid optimization methods, such as motif‐scaffolding, have been put into practice; this involves grafting evolutionarily‐validated functional motifs (e.g., viral epitopes or receptor‐binding interfaces) onto computationally optimized, hyperstable de novo scaffolds [187, 206]. For instance, the Riff‐Diff (rotamer inverted fragment finder‐diffusion) strategy successfully scaffolded complex catalytic arrays (such as the retro‐aldol tetrad) into de novo folds with atomic‐level precision (RMSD < 1 Å), achieving catalytic activities that far exceed previous pure‐synthetic attempts while maintaining extreme thermostability (folding preserved > 90°C) [188]. Similarly, in vaccine development, the I53‐50 nanoparticle platform uses genetic fusion to display the SARS‐CoV‐2 RBD in a highly ordered, multivalent array. This approach anchors a fragile, evolutionarily‐optimized viral epitope onto a computationally‐designed, 120‐subunit icosahedral core, resulting in a robust immunogen [195].

Looking ahead, the future impact of de novo protein therapeutics will depend not simply on generating novel protein structures, but on establishing a clinically actionable design paradigm that links computational innovation to therapeutic function. In tumor immunotherapy, the greatest significance of de novo design lies in its ability to move beyond the constraints of natural proteins and create molecules with programmable specificity, geometry, valency, and signaling properties, thereby opening therapeutic opportunities that are difficult to access with conventional antibodies or naturally derived cytokines. Over the next 5–10 years, the field should focus on several key priorities: developing robust frameworks to predict safety, immunogenicity, pharmacokinetics, and developability; integrating de novo design with natural scaffold optimization, directed evolution, and high‐throughput experimental screening; and building closed‐loop workflows that iteratively connect in silico modeling with biological validation. In parallel, personalized and context‐aware design strategies tailored to tumor antigens, patient genomic features, and immune states may further expand the precision and applicability of these therapeutics. Although challenges in clinical translation remain substantial, continued progress along these directions could establish de novo protein design not merely as a promising technology, but as a foundational platform for next‐generation cancer immunotherapy and precision medicine.

## Author Contributions

Jianhua Luo and Yanfeng Wu provided the idea, supervising the final draft of the manuscript. Jianhua Luo contributed to the refinement of the manuscript, suggesting structural adjustments, and providing detailed feedback on the content and presentation. Yang Jin conducted literature searching, prepared the draft, made the tables and illustrations and revised the manuscript. All authors have read and approved the final manuscript.

## Conflicts of Interest

The authors declare no conflicts of interest

## Data Availability

Not applicable.
